# Iron ore pellets based-Ag_2_O nanoparticles as efficient Bi-functional heterogeneous catalyst for the synthesis tetrahydrobenzo[α]xanthens in green media

**DOI:** 10.3389/fchem.2025.1413080

**Published:** 2025-03-19

**Authors:** Ehsan Faryabi, Enayatollah Sheikhhosseini, Mahdieh Yahyazadehfar

**Affiliations:** Department of Chemistry, Kerman Branch, Islamic Azad University, Kerman, Iran

**Keywords:** Ag_2_O NP@IOP, iron ore pellets, reusable catalyst, multi-component reaction, green synthesis, benzoxanthens

## Abstract

Through the use of a microwave, iron ore pellets (IOP)-based Ag_2_O nanoparticles were successfully synthesized. They were then characterized by means of a vibrating sample magnetometer (VSM), Brunauer-Emmett-Teller (BET) surface area analysis, energy-dispersive X-ray (EDX) analysis, powder X-ray diffraction (XRD) analysis, EDX elemental mapping, and field emission scanning electron microscopy (FESEM). High quantities of tetrahydrobenzo[a]xanthen derivatives were obtained in a brief amount of time by the newly prepared nanocomposite, known as Ag_2_O NP@IOP, in a one-pot, three-component reaction involving different aryl aldehydes, naphthol, and dimedone. There is no appreciable loss of catalytic activity when the catalyst is recycled and utilized several times, and it can be easily retrieved using an external magnet. The reason for functionality of designed hybrid catalyst can be related to textural properties such as desirable specific surface area and significant porosity as well as the structural nature of the Ag_2_O NP@IOP catalyst.

## 1 Introduction

Iron Ore pellet catalyst is one of the natural, heterogeneous and environmentally friendly catalysts obtained from iron ore. This catalyst has a natural source and is abundantly found in Iran through pelletizing factories. The natural iron pellet catalyst used in this research was provided from the southern region of Sirjan. The components of this catalyst are: Fe (%67–68), FeO (%5), SiO_2_ (%5.1), MgO (%1.2), CaO (≤%0.6), Al_2_O_3_ (≤%0.46), TiO_2_ (%0.01) and several ppm of other metals (Mn, S, C, P) and they have physical properties: brittleness strength (C.C.S = 270 kg/pellet), abradability index (A. I = %5.4), porosity (% 20–22) and size (8–16 mm).

As a result, as the materials of the iron pellet show, nearly 70 percent of the pellet is made up of iron metal, and this stable intermediate metal can act as a Lewis acid catalyst due to having empty orbitals. Although this feature can be found in its other constituent materials such as Al_2_O_3_ and TiO_2_, but these constitute a small percentage of the constituent materials. On the other hand, the iron ore pellet catalyst is insoluble in aqueous and organic solvents, therefore, after the reaction, it can be easily separated from the reaction environment and can be reused with proper washing. Also, this catalyst is well able to catalyze organic reactions in green water solvent. Therefore, iron pellets can be introduced as a natural, recyclable and environmentally friendly heterogeneous catalyst to reduce pollution related to the reaction of preparing organic heterocycles (Sheikhhosseini et al., 2016).

Employing solid support to prepare catalysts is one of the key steps in creating new heterogeneous catalysts. In the majority of these situations, solidly supported catalysts may be superior to their unsupported counterparts in terms of separation, versatility, and—most importantly—their capacity to offer useful convenience in a continuous system, which is highly prized in the sector. The features of the support play a crucial role in determining the catalytic activity of supported catalysts. Because of their high surface-to-volume ratio, environmental friendliness, reusability, straightforward work-up processes, and simplicity of separation, nanoparticles, as heterogeneous catalysts, have drawn a lot of interest as effective catalysts in many organic reactions (Collard et al., 2014; Ghasemzadeh et al., 2013; Nguyen et al., 2024; Patel et al., 2024) Particularly, magnetic nanoparticles have drawn more attention recently (Ma’mani et al., 2010) due to their usefulness in organic synthesis (Ghasemzadeh et al., 2013), the advancement of contemporary technology, including electronics and biomedicine (Ho et al., 2011), and their growing involvement in these fields.

Catalyst studies have historically focused on metal oxide semiconductor catalysts, including Ag_2_O, ZnO, Fe_2_O_3_, BiVO_4_, Bi_2_WO_6_, ZnWO_4_, etc., (He et al., 2022; Zhang et al., 2021). Critical factors, including surface characteristics and crystal structure, influence semiconductor catalyst catalytic efficiency (Ma et al., 2018). The degrading impact of the catalyst may be greatly enhanced by adjusting these parameters (Ghasemipour et al., 2020; Liu et al., 2023). Ag_2_O has a distinct electrical conductivity and is a p-type semiconductor catalyst. Ag_2_O is a semiconductor material with a simple cubic structure and a small band gap. Its minimal orbital energy and huge ion size are reported. Ag_2_O has a band gap energy of 1.2 eV and appropriate valence band (VB) and conduction band (CB) edge locations (Xu et al., 2022; Wang et al., 2021; Jung et al., 2024; Xie and Leo, 2022). While the materials are more easily activated by external stimuli, the small band gap is detrimental to the catalytic performance’s long-term survival. Pure Ag_2_O is not very stable, just as other compounds based on silver are. The experiment demonstrated that Ag_2_O will experience a breakdown process when exposed to light, which will cause a significant drop in its usable life. One efficient way to increase the stability of semiconductor catalysts is to build heterojunctions made of Ag_2_O.

**SCHEME 1:**
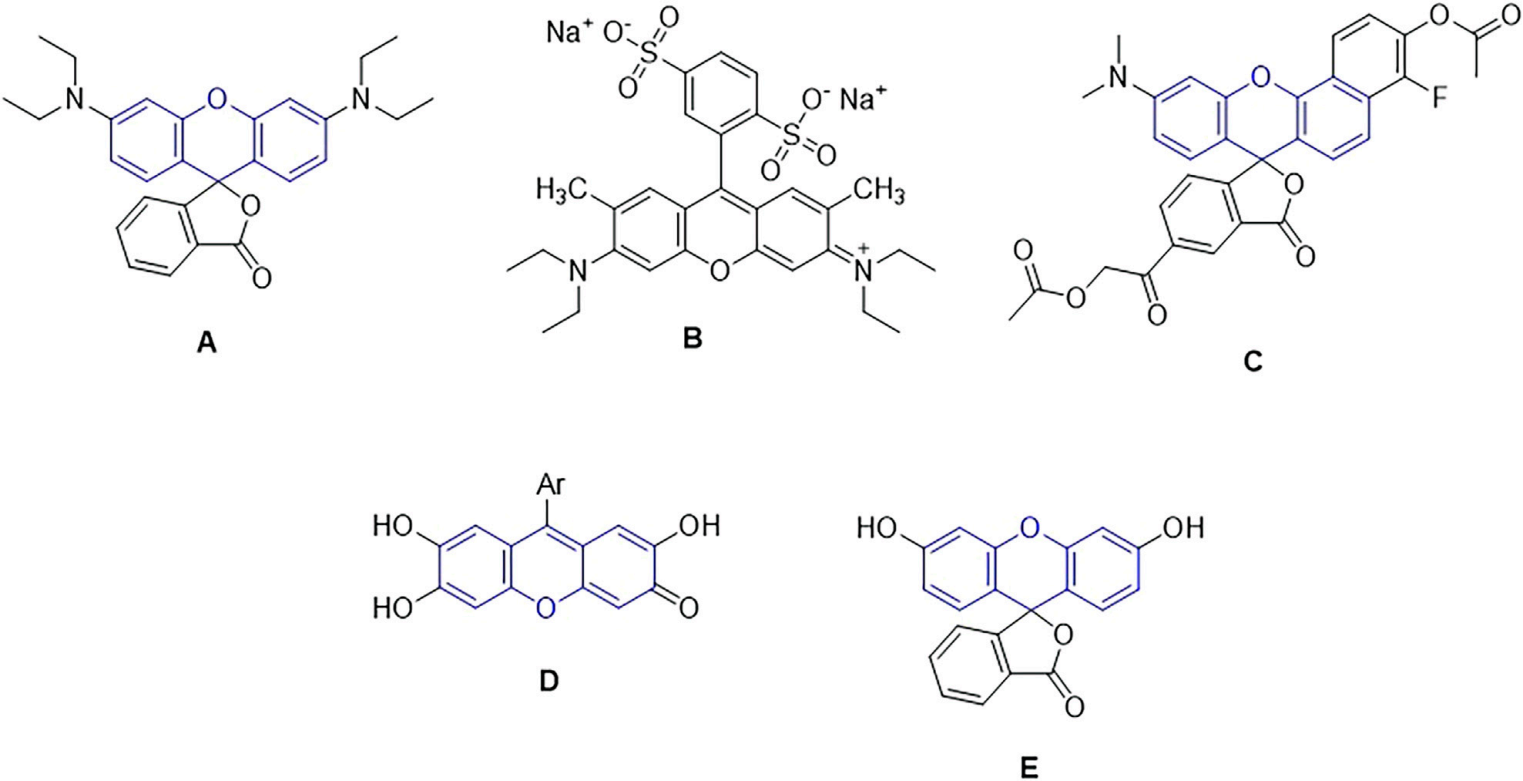
The chemical structure of important compounds based on the xanthen unit (structures (**A-E**)).

Chemists are always focused on creating heterocyclic compounds that resemble nature since they are widely distributed in the natural world. Because of their low cost of synthesis, several functionalization options (Shirini et al., 2014a), and well-established importance in medicinal chemistry (Das et al., 2007), benzoxanthones are among the most significant groups of heterocyclic molecules. A heterocyclic compound that contains oxygen, benzoxanthones have been shown to have a variety of documented properties, including antiviral, anti-diabetic, antibacterial, anti-inflammatory, antioxidant, antimalarial, anti-HIV, antiallergic, antiplatelet, anticarcinogenic, and antitumor properties (Bedi et al., 2023). In addition, they can be utilized as fluorescent materials for dyes, laser technologies, pH sensors, and other devices (Madhav et al., 2008; Bosica et al., 2023). Many advanced compounds including pharmaceuticals, natural products, and industrial compounds exist which have xanthene-based units (Scheme 1) (Saied et al., 2022). The importance of each of these compounds is as follows.

Because of their superior photostability, broad fluorescence in the visible portion of the electromagnetic spectrum, high absorption coefficient, and high fluorescence quantum yield, rhodamine dyes are frequently utilized as fluorescent probes. Rhodamines are used as pigments, fluorescence standards (for polarization (Prazeres et al., 2004a; Prazeres et al., 2008) and quantum yield (Crosby and Demas, 1971), laser dyes (Drexhage, 1976), fluorescent probes to characterize the fluidity of lipid membranes (Bogen et al., 1998), the surface of polymer nanoparticles (Farinha et al., 2001; Fonseca et al., 2007), and imaging in living cells (Liu et al., 2008), include single-molecule imaging (Li et al., 2008; Bossi et al., 2008), micelle structure and dynamics investigations (Quitevis et al., 1993), polymer-bioconjugate detection (Nicolas et al., 2006), and oligonucleotide adsorption on latex research (Prazeres et al., 2004b). Rhodamine derivatives have also been employed for surface modification of a virus, as a thermometer (Shiraishi et al., 2007; Schlick et al., 2005; Shiraishi et al., 2008), as molecular switches, (Bossi et al., 2006), thiols among other analytes (Kim et al., 2008) and particularly as chemosensors used either *in vitro* as *in vivo* in detection of Hg (II), Cu (II), Fe (III), Cr (III). Biological probes, tracer agents, and laser dyes are all common applications for rhodamine dyes and their fluorogenic derivatives. By adding various substituents to the rhodamine nitrogen’s (structure **A**), the dye’s optical characteristics may be altered, giving it a wide range of applications.

When exposed to yellow light, sodium 4-[6-(diethylamino)-3-(diethyliminio)-3H-xanthen-9-yl] benzene-1,3-disulfonate (acid red 52) acts as a photocatalyst to effectively acetalize aldehydes with alcohols at room temperature. Lately, luminescent acid red 52 and its derivatives have been widely used in a variety of fields, including fluorescent chemosensors, composite fluorescent pigments, fluorescence switches, and calf thymus deoxyribonucleic acid (CT DNA) of a fluorescent counterion, respectively (structure **B**) (Yu et al., 2020).

Long-wavelength benzo[c]-xanthene dyes known as seminaphthorhodafluors (SNARFs) are a generic family of fluorescent indicators that have been described for pH monitoring in both excitation and emission ratio applications (Dual Emission pH Sensors). These intracellular pH markers have been used extensively in recent years to track intracellular pH variations (structure **C**) (Whitaker et al., 1991).

Derivatives of synthetic 9-aryl substituted xanthene-3-one have antimicrobial, antibacterial, antifungal, and antiproliferative properties against tumor cell lines, as well as an affinity for binding enzymes (structure **D**) (Veljovic et al., 2022).

A variety of physiologically significant analytes may be detected using fluorescein, a reporter molecule that is commonly employed (structure **E**) (Kowada et al., 2015).

Several procedures, including the cyclocondensation reaction involving 2-hydroxy aromatic aldehydes and 2-tetralone, are known in the literature for the production of benzo[α]xanthen derivatives using multicomponent systems (Shirini et al., 2014b; Jha and Beal, 2004). The condensation of an activated methylene source, an aldehyde, and an aromatic nucleophile like *β*-naphthol is one of the most significant processes frequently employed for this reason (Pirouzmand et al., 2017; Imon et al., 2022; Chaskar et al., 2011; Kheirkhah et al., 2018).

The use of heterogeneous and homogeneous catalysts for benzo[α]xanthen synthesis has been documented in an increasing number of articles in recent years. This is because these catalysts are easily recyclable, which reduces waste creation, and they typically produce a safe and clean synthetic process. Some examples include the use of [Fe_2_O_3_@HAp]-supported dual acidic nanocatalyst (Kheirkhah et al., 2018), [DSTMG][CF_3_COO] (Dutta et al., 2017), [CTA]Fe/MCM-41(DS) (Pirouzmand et al., 2017), BF_3_:OEt_2_ and EtOH (Sethukumar et al., 2011) and IBX (Chaskar et al., 2011). All of the documented synthetic procedures used to produce xanthen ones use expensive, hard-to-find heterogeneous catalysts, and the majority of them fail to provide moderate to good yields, even after prolonged reaction times.

In this research, the attempt to use the natural substrate of iron pellets for the stability of Ag_2_O nanoparticles led to the synthesis of a new Ag_2_O NP@IOP nanocatalyst. The synthesis of tetrahydrobenzo[α]xanthen derivatives from aromatic aldehydes, dimedone, and *β*-naphthol was then studied using this active catalyst in a three-component process.

## 2 Experimental section

### 2.1 Chemicals and reagent

Dimedone, *β*-naphthol, aromatic aldehydes, silver nitrate and sodium hydroxide were provided from Merck company. Each material has been of analytical or synthetic grade with the increased purity. The natural iron pellet catalyst used in this research was provided from the southern region of Sirjan.

### 2.2 Material characterization

Every XRD result was gathered using a Philips PC-APD X-ray diffractometer (XRD, Netherlands). Energy Dispersive Spectroscopy (SEM-EDS analysis; EM 3200 SEM and KYKY; China) was utilized to analyze the heterogeneous catalyst. A thermoanalyzer (TG 209F3 NETZSCH) was used to analyze thermal behavior in N_2_ between ambient temperature and 350°C. A TriStar II Plus surface area and porosity analyzer operating at 77 K was used to quantify N_2_ adsorption-desorption isotherms (BET). Magnetization measurements were carried out with a Lakeshore (model 7,407) under magnetic fields at room temperature. The purity of the reactants and reaction progress were checked by thin layer chromatography (TLC) on aluminum-backed plates precoated with 0.25 mm E-Merck silica gel 60- F_254_ silicagel. Melting points were uncorrected and measured by Electro thermal 9,100 apparatus in open capillary tubes. IR spectra were recorded on a JASCO FT-IR-4000 spectrophotometer operating (range of 400–4,000 cm^−1^) with KBr pellets. ^1^H and ^13^C NMR spectra were also obtained with a Bruker AC (250 MHz for ^1^H NMR and 62.5 MHz and ^13^C NMR) in DMSO-*d*_*6*_ as solvents and tetramethylsilane (TMS) as the internal reference.

### 2.3 Fabrication of nanocatalyst

#### 2.3.1 Fabrication of Ag_2_O nanoparticles

Ag_2_O nanoparticles were synthesized using the standard co-precipitation method from aqueous solutions of sodium hydroxide (NaOH) and silver nitrate salt. First, 80 mL of distilled water were used to produce 0.005 M silver nitrate. Drop by drop, the NaOH solution (0.025 M) was combined with the silver nitrate until the pH reached 11. After that, the reaction mixture was continually stirred at 60°C for 2 hours. The end result was the development of grayish Ag_2_O precipitates. After that, distilled water was used to wash these precipitates until the pH reached 7, and then they were permitted to dry overnight (Sullivan et al., 2013).

#### 2.3.2 Fabrication of Ag_2_O NP@IOP nanocatalysts as heterogeneous catalyst

**SCHEME 2:**
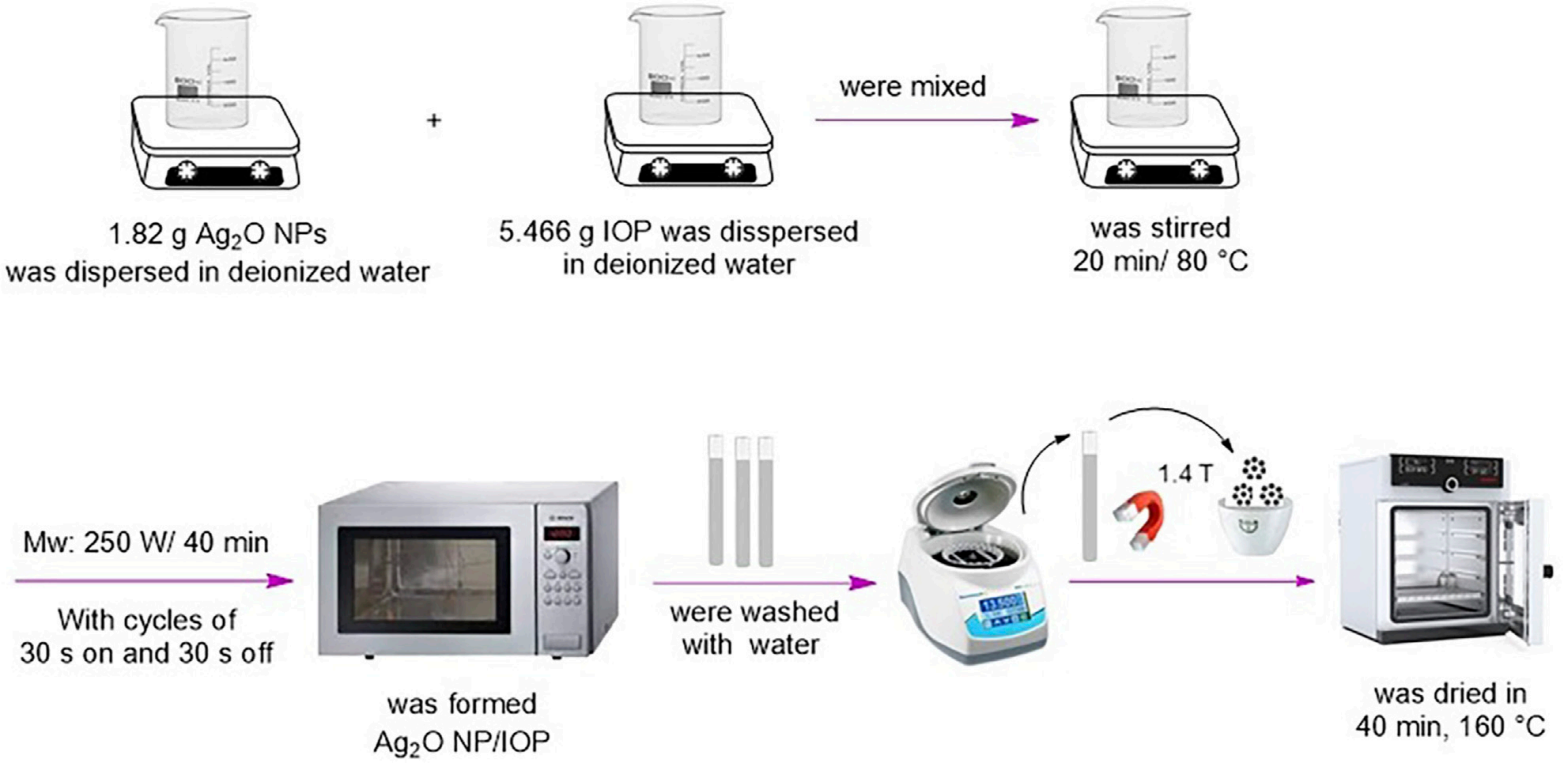
Synthesis of Ag_2_O NP@IOP nanocatalyst.

1.822 g of Ag_2_O nanoparticles and 5.466 g of IOP were dispersed in deionized water using a magnetic stirrer for 20 min. The mixture was transferred to a microwave oven (250 W) and microwaved for 40 min. After that, the final product was dried for 40 min at 160°C and washed with distilled water (Scheme 2).

### 2.4 General process of preparing tetrahydrobenzo[α]xanthen derivatives using Ag_2_O NP@IOP nanocatalyst

At 40°C, a mixture of 3.0 mmol aromatic aldehyde, 3.0 mmol β-naphthol, 3.0 mmol 5,5-dimethyl-1,3-cyclohexanedione, and 20 w.t.% (0.094 g) Ag_2_O NP@IOP was stirred. TLC was used to monitor the progression of the reaction. After the reaction was complete, the solid product was dissolved in acetone, and the catalyst was removed from the mixture with an external magnet. To reuse it for the subsequent reaction, the catalyst was repeatedly washed with acetone and water and dried in an oven.

### 2.5 Selected spectral data

***12-(2-hydroxy-3-methoxyphenyl)-9,9-dimethyl-8,9,10,12-tetrahydro-11H-benzo[a]xanthen-11-one (4a)*:** Yield: 94%. M.p. = 211–212°C. ^1^H NMR (250 MHz, CDCl_3_): δ = 0.98 (s, 3H, CH_3_), 1.10 (s, 3H, CH_3_), 1.96 (brs, 2H, CH_2_), 2.33 (d, *J* = 5.75 Hz, 1H, CH_2_), 2.58 (d, *J* = 10 Hz, 1H, CH_2_), 3.85 (s, 3H, OCH_3_), 4.67 (s, 1H, CH), 6.57–6.91 (m, 7H, H-Ar), 7.27–7.36 (m, 1H, H-Ar), 7.73–7.80 (m, 1H, H-Ar), 10.41 (brs, 1H, OH). ^13^C NMR (62.5 MHz, CDCl_3_): δ = 26.2, 26.8, 38.6, 40.2, 47.0, 47.6, 53.1, 107.4, 107.94, 113. 7, 115.2, 116.8, 117.8, 120.7, 121.5, 122.3, 124.4, 125.2, 126.0, 137.6, 137.9, 144.1, 144.5, 165.7, 167.8, 198.3.

***12-(3-hydroxynaphthalen-2-yl)-9,9-dimethyl-8,9,10,12-tetrahydro-11H-benzo[a]xanthen-11-one (4b):*** Yield: 99%. M,p. = 260–263°C. IR (KBr, cm^−1^): 3,175, 2,946, 2,892, 2,863, 1,644, 1,594, 1,373, 1,011–1,333, 791–980. ^1^H NMR (250 MHz, CDCl_3_): δ = 1.05 (s, 3H, CH_3_), 1.15 (s, 3H, CH_3_), 1.88 (d, *J* = 18 Hz, 1H, CH_2_), 2.39 (brs, 2H, CH_2_), 2.60 (d, *J* = 9.25 Hz, 1H, CH_2_), 5.25 (s, 1H, CH), 7.26–7.45 (m, 6H, H-Ar), 7.72 (brs, 6H, H-Ar), 10.68 (brs, 1H, OH). ^13^C NMR (62.5 MHz, CDCl_3_): δ = 26.4, 26.9, 38.4, 40.2, 47.0, 47.8, 108.1, 112.8, 113.2, 113.6, 114.4, 114.7, 119.9, 120.6, 121.7, 122.5, 123.0, 123.2, 123.7, 124.4, 125.5, 126.4, 128.0, 128.3, 129.0, 145.9, 166.1, 167.3, 198.1.

***12-(5-bromo-2-hydroxyphenyl)-9,9-dimethyl-8,9,10,12-tetrahydro-11H-benzo[a]xanthen-11-one (4c)***: Yield: 98%. M,p. = 248–250°C. ^1^H NMR (DMSO-*d*_*6*_, 250 MHz): δ = 0.91 (s, 6H, 2 CH_3_), 2.06–2.47 (m, 4H, 2CH_2_), 5.01 (s, 1H, CH), 6.62–6.65 (m, 1H, H-Ar), 6.99–7.40 (m, 5H, H-Ar), 7.83–7.85 (m, 2H, H-Ar), 8.21–8.24 (m, 1H, H-Ar), 9.99 (brs, 1H, OH).

***9,9-dimethyl-12-(p-tolyl)-8,9,10,12-tetrahydro-11H-benzo[a]xanthen-11-one (4d)***: Yield: 95%. M,p. = 176–177°C. IR (KBr, cm^−1^): 3,175, 2,946, 2,892, 2,863, 1,644, 1,594, 1,373, 1,011–1,333, 791–980. ^1^H NMR (250 MHz, DMSO-*d*_*6*_): δ = 0.85 (s, 3H, CH_3_), 1.02 (s, 3H, CH_3_), 2.10 (s, 3H, CH_3_), 2.27–2.57 (m, 4H, 2CH_2_), 5.49 (s, 1H, CH), 6.95–7.98 (m, 10H, H-Ar).

***12-(2-methoxyphenyl)-9,9-dimethyl-8,9,10,12-tetrahydro-11H-benzo[a]xanthen-11-one (4e)*:** Yield: 98%. M,p. = 168–169°C. ^1^H NMR (250 MHz, DMSO-*d*_*6*_): δ = 0.82 (s, 3H, CH_3_), 1.07 (s, 3H, CH_3_), 2.02 (d, *J* = 20 Hz, 2H, CH_2_), 2.21 (brs, 2H, CH_2_), 3.70 (s, 3H, OCH_3_), 5.81 (s, 1H, CH), 6.67–7.03 (m, 10 H, H-Ar).

**12-(4-methoxyphenyl)-9,9-dimethyl-8,9,10,12-tetrahydro-11H-benzo[a]xanthen-11-one *(4f)*:** Yield: 98%. M,p. = 204–207°C. ^1^H NMR (250 MHz, DMSO-*d*_*6*_): δ = 0.86 (s, 3H, CH_3_), 0.99 (s, 3H, CH_3_), 2.05–2.48 (m, 4H, 2CH_2_), 3.64 (s, 3H, OCH_3_), 4.41 (s, 1H, CH), 6.73–7.04 (m, 10 H, H-Ar).

***12-(4-(dimethylamino)phenyl)-9,9-dimethyl-8,9,10,12-tetrahydro-11H-benzo[a]xanthen-11-one (4g):*** Yield: 99%. M,p. = 195–197°C. ^1^H NMR (250 MHz, DMSO-*d*_*6*_): δ = 1.01 (s, 6H, 2CH_3_), 2.29 (brs, 2H, CH_2_), 2.79 (brs, 2H, CH_2_), 3.26 (s, 6H, 2CH_3_), 5.73 (s, 1H, CH), 6.55–6.76 (m, 10 H, H-Ar).

***9,9-dimethyl-12-(4-nitrophenyl)-8,9,10,12-tetrahydro-11H-benzo[a]xanthen-11-one (4h)*:** Yield: 90%. M,p. = 175–177°C. ^1^H NMR (250 MHz, DMSO-*d*_*6*_): δ = 0.83 (s, 3H, CH_3_), 1.01 (s, 3H, CH_3_), 2.06 (d, *J* = 20 Hz, 2H, CH_2_), 2.32 (brs, 2H, CH_2_), 5.73 (s, 1H, CH), 7.18 (d, *J* = 5 Hz, 2H, H-Ar), 7.32 (d, *J* = 5 Hz, 2H, H-Ar), 7.41 (d, *J* = 7.5 Hz, 2H, H-Ar), 7.57 (d, *J* = 7.5 Hz, 2H, H-Ar), 8.04 (t, *J* = 7.5 Hz, 2H, H-Ar).

***9,9-dimethyl-12-(3-nitrophenyl)-8,9,10,12-tetrahydro-11H-benzo[a]xanthen-11-one (4i)*:** Yield: 97%. M,p. = 169–173°C. ^1^H NMR (250 MHz, DMSO-*d*_*6*_): δ = 1.02 (s, 6H, 2CH_3_), 2.06–2.46 (m, 4H, 2CH_2_), 5.77 (s, 1H, CH), 6.99–7.08 (m, 2H, H-Ar), 7.47–7.94 (m, 8H, H-Ar).

***12-(2,4-dimethoxyphenyl)-9,9-dimethyl-8,9,10,12-tetrahydro-11H-benzo[a]xanthen-11-one (4j)*:** Yield: 98%. M,p. = 192–195°C. ^1^H NMR (250 MHz, DMSO-*d*_*6*_): δ = 0.95 (s, 6H, 2CH_3_), 2.01–2.47 (m, 4H, 2CH_2_), 3.88 (s, 6H, 2OCH_3_), 5.69 (s, 1H, CH), 6.27–6.72 (m, 9H, H-Ar).

***9,9-dimethyl-12-(3,4,5-trimethoxyphenyl)-8,9,10,12-tetrahydro-11H-benzo[a]xanthen-11-one (4k)*:** Yield: 99%. M,p. = 198–201°C. ^1^H NMR (250 MHz, DMSO-*d*_*6*_): δ = 1.03 (s, 6H, 2CH_3_), 2.06–2.48 (m, 4H, 2CH_2_), 3.62 (s, 9H, 3OCH_3_), 5.82 (s, 1H, CH), 6.22–6.41 (m, 6H, H-Ar), 6.76 (brs, 1H, H-Ar), 6.86–6.87 (m, 1H, H-Ar).

***12-(4-hydroxy-3-methoxyphenyl)-9,9-dimethyl-8,9,10,12-tetrahydro-11H-benzo[a]xanthen-11-one (4l)*:** Yield: 98%. M,p. = 194–197°C. ^1^H NMR (250 MHz, DMSO-*d*_*6*_): δ = 1.00 (s, 6H, 2CH_3_), 2.28 (brs, 4H, 2CH_2_), 3.84 (brs, 3H, OCH_3_), 5.72 (s, 1H, CH), 6.50 (brs, 9H, H-Ar), 8.59 (brs, 1H, H-Ar).

## 3 Results and discussion

### 3.1 Characterization and synthesis of Ag_2_O NP@IOP nanocatalyst

**FIGURE 1:**
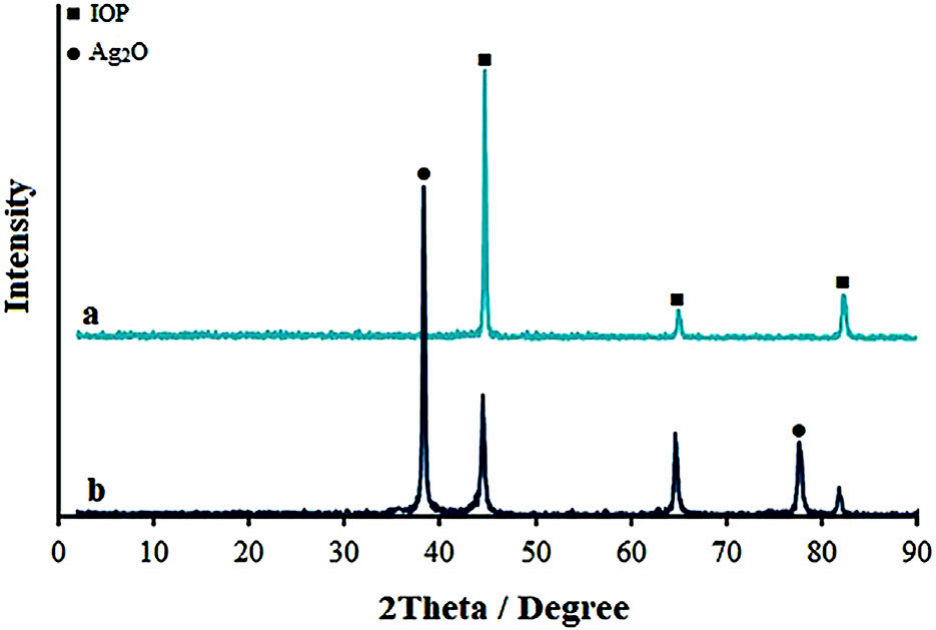
XRD pattern of **(A)** IOP, and **(B)** Ag_2_O NP@IOP nanocomposite.

The crystal structures of IOP and Ag_2_O Ag_2_O NP@IOP nanocatalyst were characterized by XRD (Figure 1). In the spectrogram of the IOP sample, the main diffraction peaks at *2θ* of 44.8°, 65.1°, and 82.5°, which proved that IOP crystals belonging to the cubic system (JCPDS No.06-0696) were successfully characterized. In the XRD spectrum of the Ag_2_O NP@IOP nanocatalysts, the presence of Ag_2_O was proved by the characteristic diffraction peak at *2θ* of 38.2° and 77.4°, which corresponded to the (200) and (400) crystal faces of Ag_2_O cubic crystal (JCPDS no. 00–012–0793) (Warsi et al., 2022). According to the calculation formula of crystal plane spacing of cubic crystal system and Bragg equation, the crystal face (200) corresponding to 38.2° was selected, and a = b = c = 4.086 Å of Ag_2_O was calculated. The crystallite size for the synthesized nanocomposite is calculated using Debye-Scherer Equation 1:

$$D=\frac{0.9\lambda }{\beta \mathrm{Cos}\theta }$$ (1)

Where λ is the wavelength of X-ray and *β* is full width at half maximum of the peak at diffracting angle *θ*. Based on the most prominent characteristic peak for the (200) diffraction plane, the calculated crystallite size was 38.5 nm for Ag_2_O nanoparticles.

**FIGURE 2:**
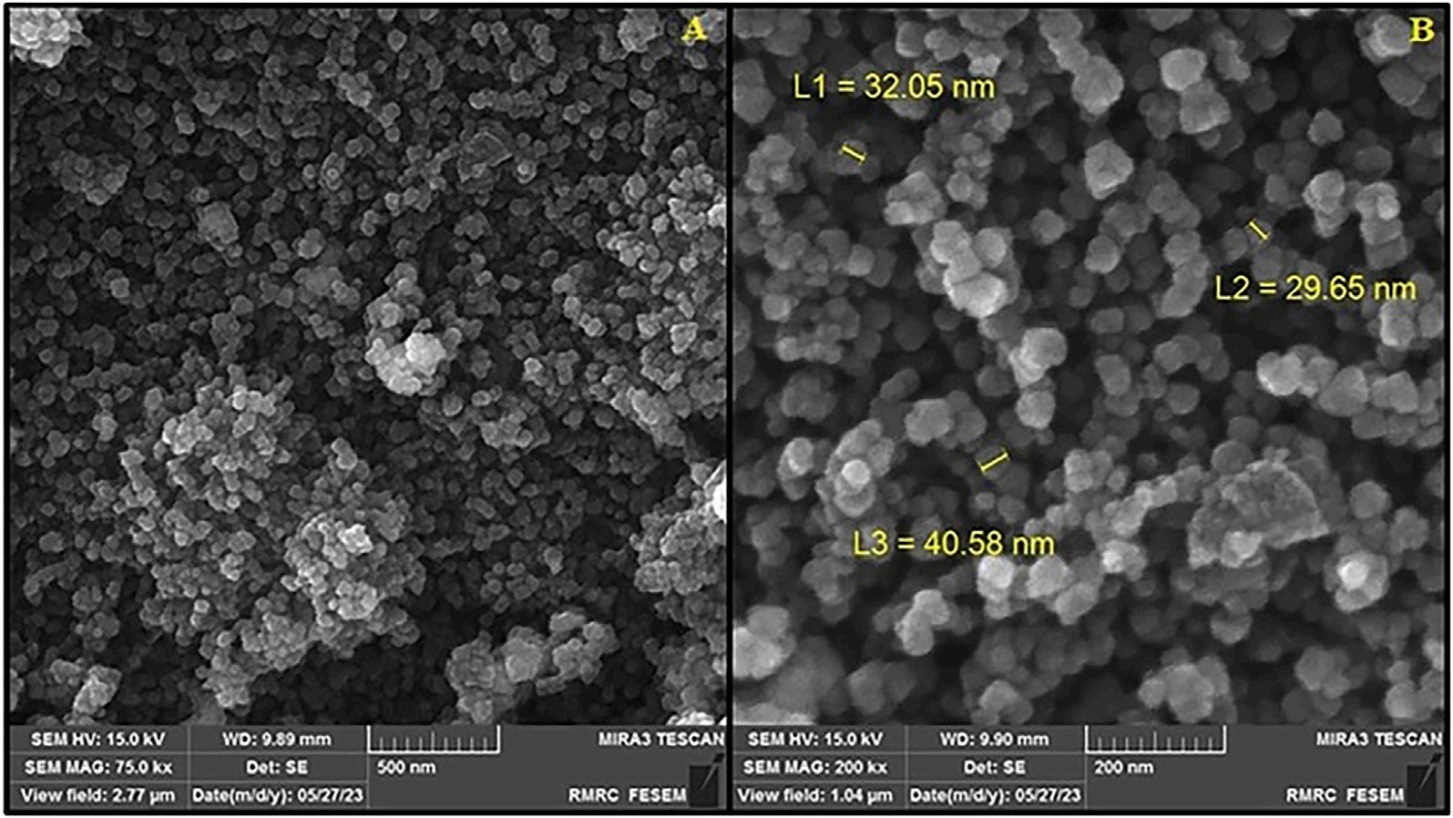
**(A)** FESEM image, and **(B)** High resolution FESEM image of Ag_2_O NP/IOP nanocatalyst.

In order to study its morphology, the morphology of Ag_2_O NP/IOP nanocatalyst were characterized by SEM. The SEM image in Figure 2 showed that the morphology of nanocomposite was mainly composed of nano spherical particles with a diameter of about 20–40 nm with regular morphology.

**FIGURE 3:**
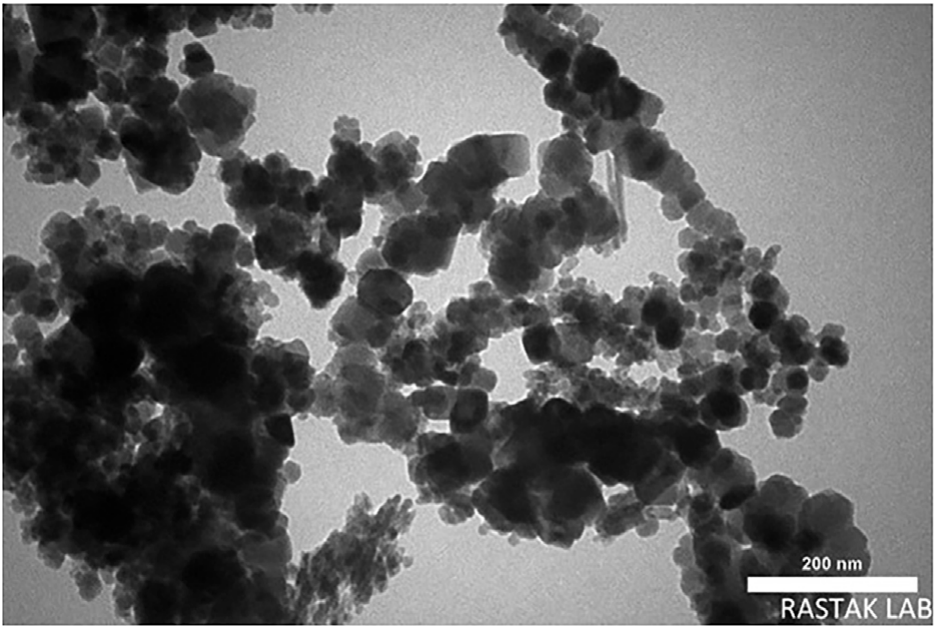
TEM image of Ag_2_O NP/IOP nanocatalyst synthesized by microwave method.

Figure 3 depicts a TEM picture of Ag_2_O NP@IOP nanocatalyst synthesized using microwave technique. According to this figure, the catalyst has a spherical shaped morphology with narrow particle size distribution. Furthermore, based on the TEM image, the shape (morphology) of the Ag_2_O NP@IOP sample is homogenous, indicating the stability of the synthesized catalyst for potential application.

**FIGURE 4:**
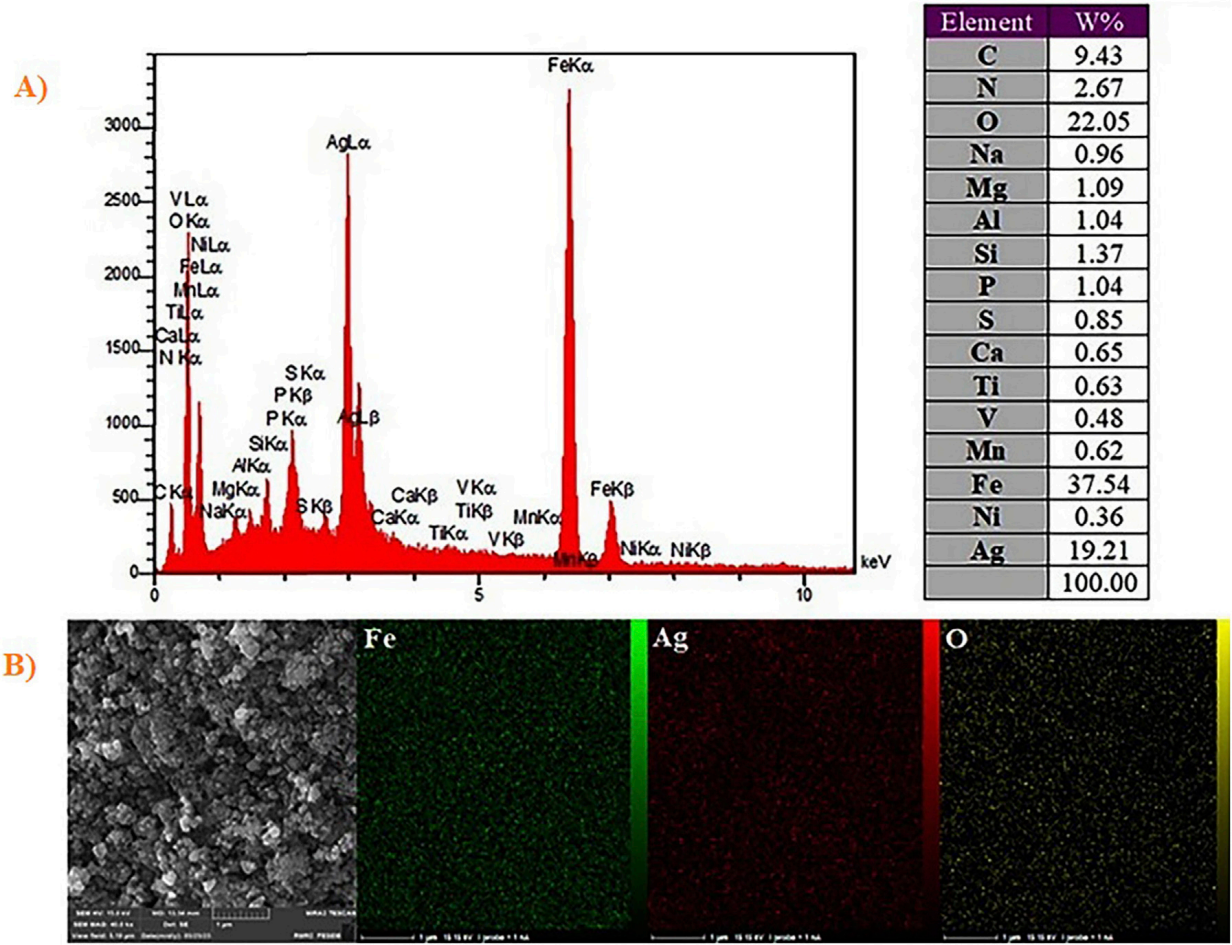
**(A)** EDX spectra of Ag_2_O NP@IOP nanocatalyst, and **(B)** Elemental mapping of Ag_2_O NP@IOP nanocatalyst.

Elemental analyses have been conducted by EDX for confirming that nanocomposites have been fabricated by Fe, Ag, O, V, Ni, Mn, Ti, K, Al, Mg, Na, S, P and C. In order to select regions with greater contents of Fe and Ag, mapping and scanning have been conducted on a specimen (Figures 4A, B). According to Figure 4B, elemental map, which results from super-position of each element (Fe, Ag and O), has been determined in the chosen region. These supports forming nanocomposite of Ag_2_O NP@IOP.

**FIGURE 5:**
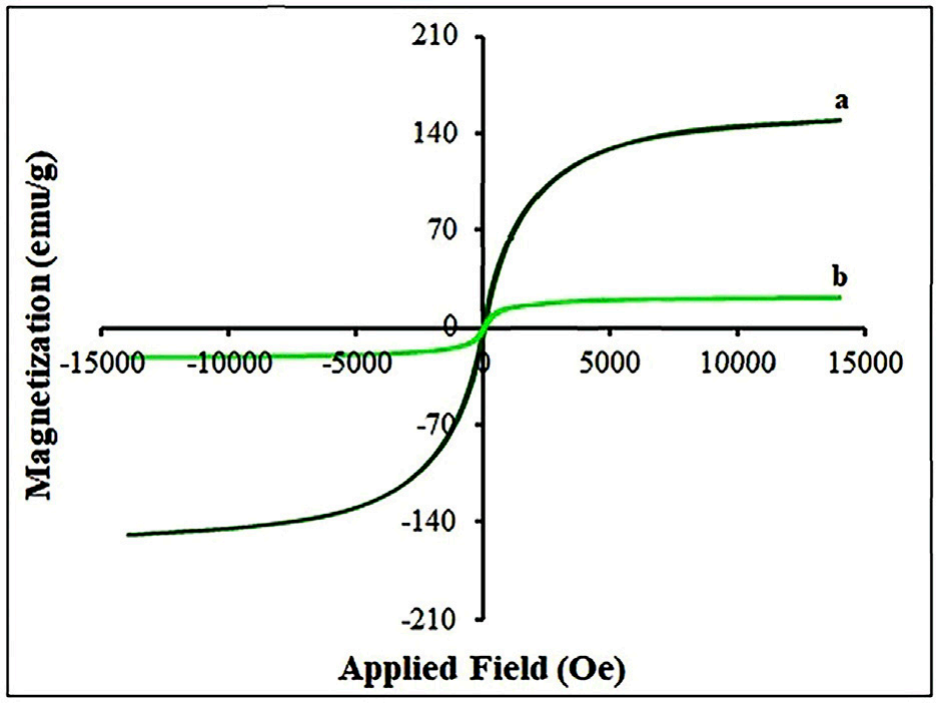
VSM magnetization curves of **(A)** IOP, and **(B)** Ag_2_O NP@IOP nanocatalyst.

The magnetization of the sample was analyzed using a vibratory sample magnetometer (VSM). The VSM analysis generated a hysteresis loop, emphasizing saturation magnetization (Ms). The Ms is defined as the maximum magnetic moment that can be induced in the material under the influence of an external magnetic field. The IOP sample showed a Ms of 140.01 emu/g Figure 5 (curve a). On the other hand, the nanocatalyst sample displayed a fewer Ms value of 20.76 emu/g, as shown in Figure 5 (curve b). With the increase in Ag_2_O in the nanocatalyst, the magnetic component is estimated to be relatively lower which results in a decrease in the full magnetization value. When the magnetite sample is covered by Ag_2_O, the interaction between particles will be weakened so that the aggregation between particles also decreases, so the coercivity (Hc) and magnetization of magnetite IOP material will also decrease, in the formation of Ag_2_O NP@IOP nanocatalyst.

**FIGURE 6:**
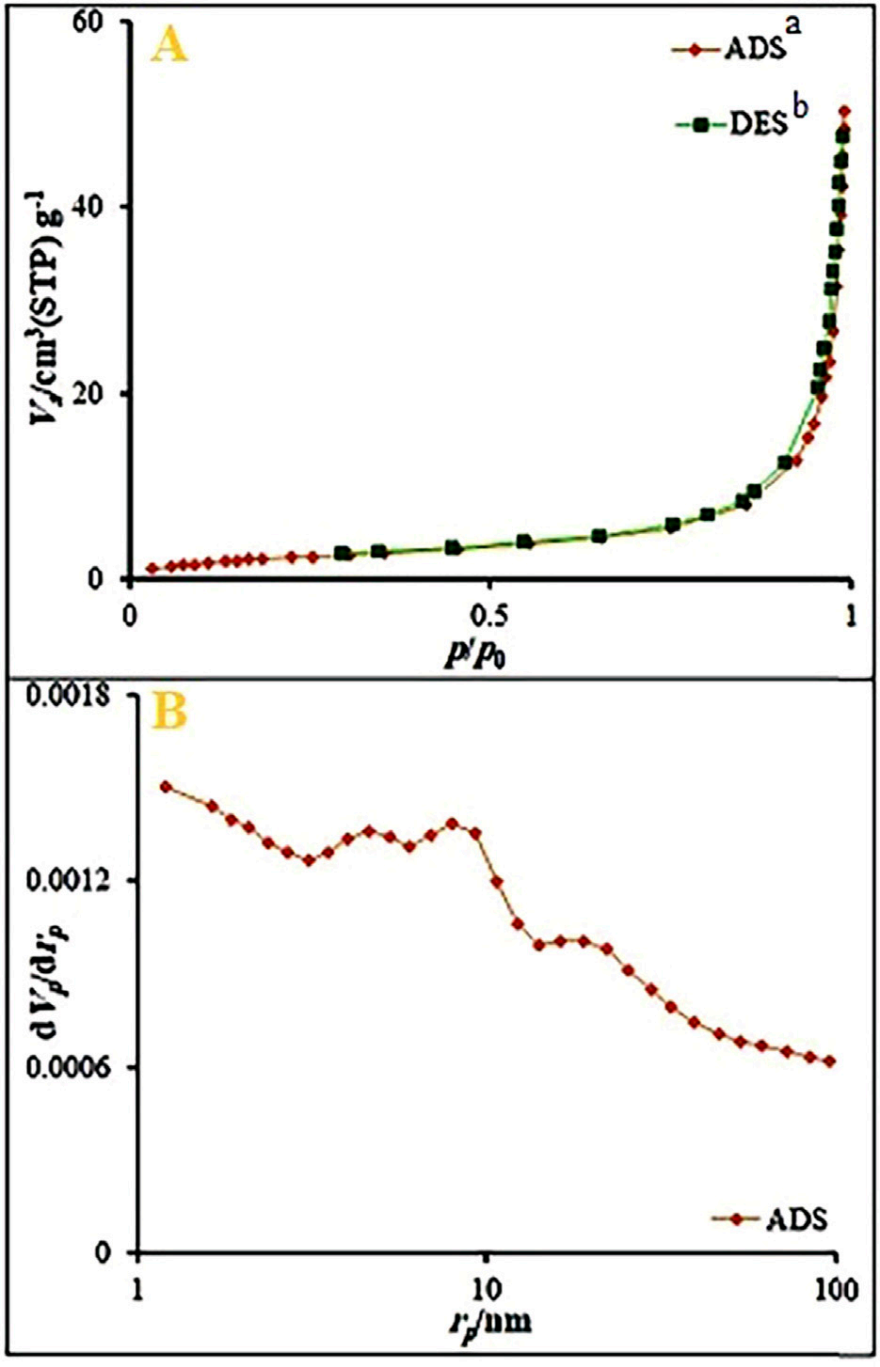
**(A)** N_2_ adsorption-desorption isotherms, and **(B)** BJH results obtained for Ag_2_O NP@IOP nanocatalyst. ^a^ADS: Adsorption. ^b^DES: Desorption.

The specific surface area was studied using the N_2_ sorption isotherms of Ag_2_O NP@IOP nanocatalyst. The results fit the type III adsorption isotherm well (Figure 6). The specific surface area of the nanocatalyst and the total volume of pores were 9.08 m^2^/g and 2.08 cm^3^/g, respectively. Using BJH (Barrett-Joyner-Halenda) study, the mesopore volume of the nanocatalyst was determined to be 0.078 cm^3^/g. The average mesopore pore diameter was 34.23 nm. This enhanced surface area of nanocatalyst could be due to a homogeneous coating and penetration of an Ag_2_O nanoparticles into the IOP intergallery which tends to expand the interspacing and thus increase the surface area.

**FIGURE 7:**
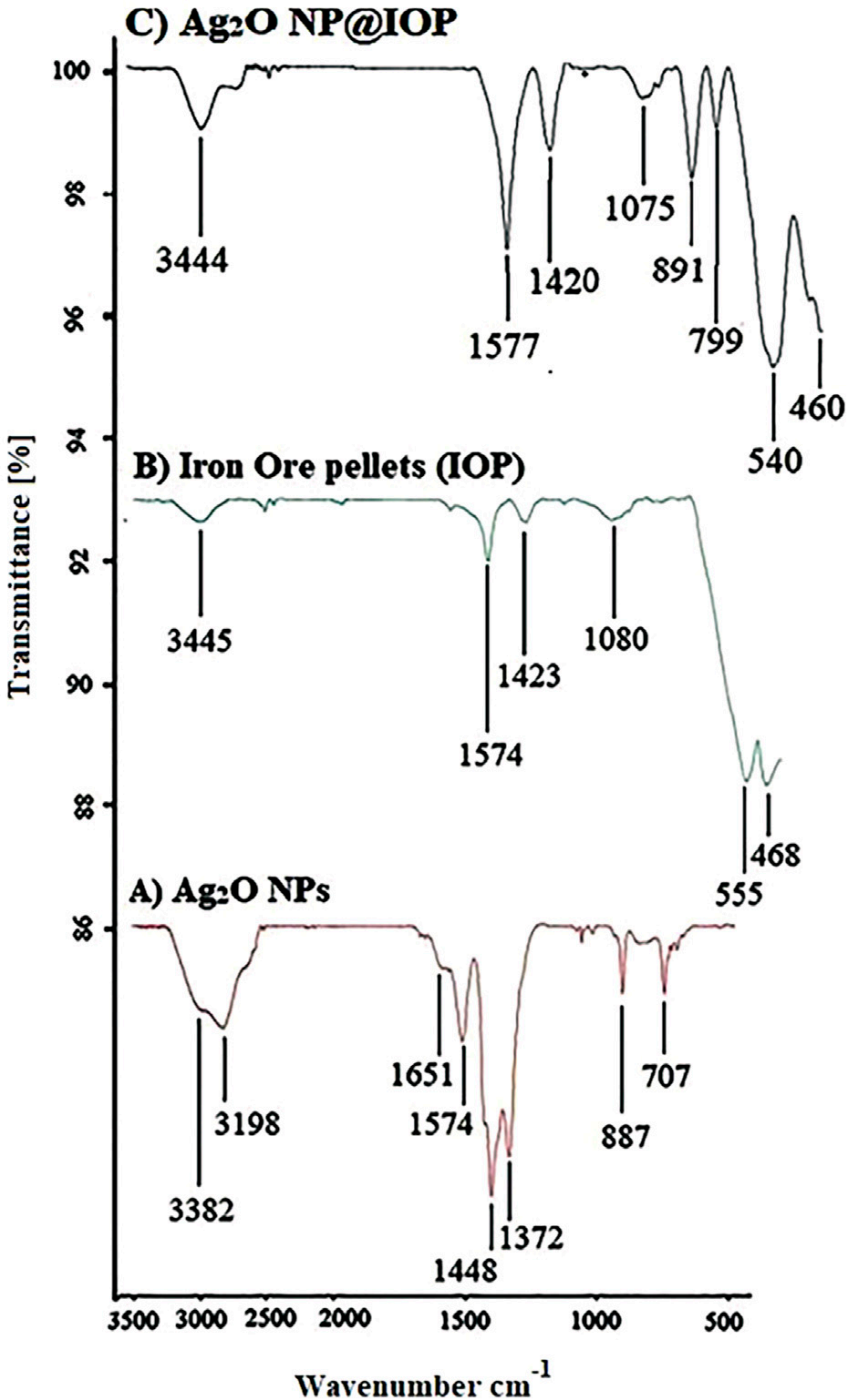
IR (KBr, υ/cm^−1^) curve of **(A)** Ag_2_O, **(B)** Iron Ore pellets (IOP), and **(C)** Ag_2_O NP@IOP.

The FT-IR of Ag_2_O, iron ore pellets (IOP) and Ag_2_O NP@IOP were shown in Figures 7A–C. In the IR spectrum of Ag_2_O nanoparticles, peaks in the 707 and 877 cm^-1^ are owned by Ag-O bending vibrations, the absorption peak in the 1,574 cm^-1^ area is for Ag-OH bending vibrations. One of the requirements of the microbial properties of silver nanoparticles is the presence of water on the surface of the silver nanoparticles, and in the FT-IR spectrum of silver nanoparticles (curve A), three peaks are found for water absorbed at the surface of the nanoparticles. The absorption peaks in 1,651, 3,198, and 3,382 cm^−1^ respectively, are attributed to the O-H stretch of free hydroxyl groups, O-H with intermolecular hydrogen bonding and to the out-of-plate bending vibration of O-H absorbed water molecules at the surface of the nanoparticles. The absorption peaks observed in the areas of 1,448 cm^−1^ and 1,372 cm^−1^ belong to the residual stretching vibrations ( ${NO}_{3}^{-1}$ ) among the silver oxide nanoparticles (Haq et al., 2021; Dasaradhudu and Srinivasan, 2020).

In the IR spectrum of iron ore pellets (IOP) (curve B), the absorption bond of 3,445 cm^−1^ is for O-H stretching vibrations. Also, the peaks in arounds of 1,080, 1,423, and 1,574 cm^−1^ respectively belong to the Fe-OH vibrations of the iron ore pellet (IOP), nitrate ( ${NO}_{3}^{-1}$ ) and Si-O-Si asymmetric stretching vibrations. The absorptions around 468 cm^−1^ and 555 cm^−1^ indicate the presence of Fe-O vibration in IOP.

In the IR spectrum of catalyst (curve C), in addition to observing the absorption of 3,444, 1,577, 1,420, 1,075, 540, and 460 related to iron pellets, partial displacement of the chemical shift of the Ag-O bonding vibrations from 877 cm^−1^ and 707 cm^−1^ in nanoparticles to 891 cm^−1^ and 799 cm^−1^ in FT-IR spectrum of the catalyst indicates the loading of silver nanoparticles on the iron pellet and interacting between them.

### 3.2 Preparation of tetrahydrobenzo[α]xanthen derivatives using Ag_2_O NP@IOP nanocatalyst

**SCHEME 3:**
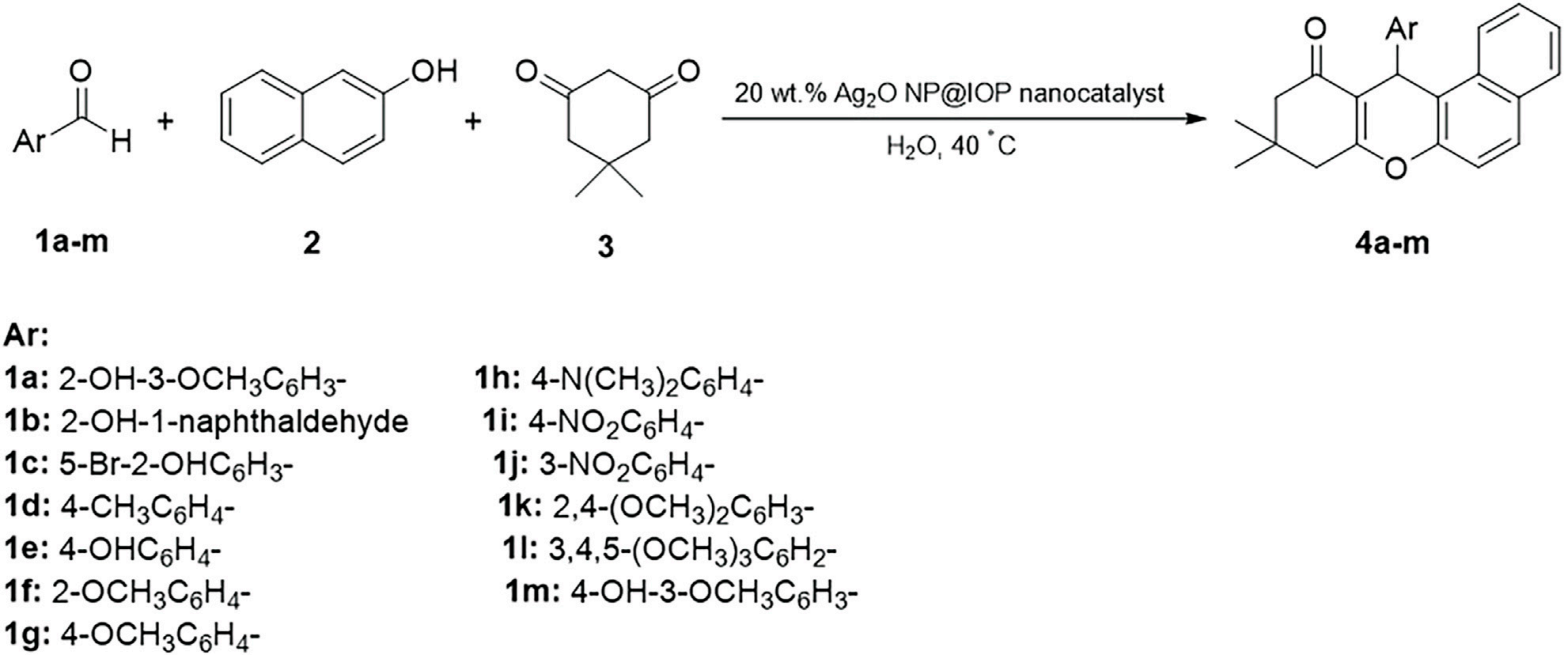
Three-component and one-pot Preparation of tetrahydrobenzo[α]xanthen-11-ones in the presence of Ag_2_O NP@IOP nanocatalyst.

Tetrahydrobenzo[α]xanthen compounds were synthesized from different aromatic aldehydes, dimedone, and *β*-naphthol in water at 40°C in presence of the Ag_2_O NP@IOP nanocatalyst. Reaction process and completion time were evaluated using thin-layer chromatography (TLC). Upon completion of the reaction, the obtained precipitates were filtered. Then, in order to separate and recover the catalyst, the produced precipitate was dissolved in acetone and an external magnet was devised to collect the catalyst particles. Following an overflow and evaporation of the solvent, the target product was achieved, which could be purified by washing with water for only once (Scheme 3).

A very important stage in any process is the optimization of reaction conditions such as the solvent type, catalyst dosage, and temperature. For this purpose, 4-nitrobenzaldehyde, *β*-naphthol, and dimedone at molar ratio of 1:1:1 was considered as the reaction model, and the reaction was evaluated under various sets of conditions in terms of solvent type, catalyst dosage, and reaction temperature. The results are presented in Table 1. To determine the optimal solvent, the reaction model was performed with different solvents, namely ethanol, methanol, ethanol: water, methanol: water, acetonitrile, dichloromethane, toluene, and polyethylene glycol in presence Ag_2_O NP@IOP at 10 wt% in reflux conditions. Accordingly, the highest efficiency and the shortest reaction time were obtained with water as solvent (Table 1, entry 5). Following with the research, the Ag_2_O NP@IOP catalyst dosage was optimized by repeating the reaction model in reflux conditions in presence of the catalyst at different dosages, namely 5, 10, 15, 20, and 25 wt%. The product could be obtained at highest efficiency within shortest reaction time when the catalyst dosage was 20 wt% (Table 1, entry 12). It was further figured out that increasing the catalyst dosage from 5 to 20 wt% effectively contributes to increased reaction rate although increasing the dosage above 20 wt% can neither increase the efficiency nor reduce the reaction time.

**TABLE 1:** Optimization of reaction conditions for the preparation of tetrahydrobenzo[α]xanthen-11-one derivatives in the presence of Ag_2_O NP@IOP nanocatalyst.

Entry^a^	Catalyst	Solvent	Tem (°C)	Time (min/h)	Yield (%)^2^
1	Ag_2_O NP@IOP 0.1 %w.t	EtOH	Reflux	15 min	73
2	Ag_2_O NP@IOP 0.1 %w.t	CH_3_OH	Reflux	13 min	77
3	Ag_2_O NP@IOP 0.1 %w.t	EtOH: H_2_O	Reflux	13 min	76
4	Ag_2_O NP@IOP 0.1 %w.t	CH_3_OH: H_2_O	Reflux	11 min	79
5	Ag_2_O NP@IOP 0.1 %w.t	H_2_O	Reflux	11 min	82
6	Ag_2_O NP@IOP 0.1 %w.t	CH_2_Cl_2_	Reflux	25 min	51
7	Ag_2_O NP@IOP 0.1 %w.t	CH_3_CN	Reflux	60 min	42
8	Ag_2_O NP@IOP 0.1 %w.t	DMF	Reflux	50 min	45
9	Ag_2_O NP@IOP 0.1 %w.t	C_6_H_5_CH_3_	Reflux	6 min	40
10	Ag_2_O NP@IOP 0.1 %w.t	PEG	Reflux	40 min	50
11	Ag_2_O NP@IOP 0.25 %w.t	H_2_O	Reflux	3 min	98
12	Ag_2_O NP@IOP 0.2 %w.t	H_2_O	Reflux	3 min	98
13	Ag_2_O NP@IOP 0.15 %w.t	H_2_O	Reflux	8 min	91
14	Ag_2_O NP@IOP 0.2 %w.t	H_2_O	40	3 min	98
15	Ag_2_O NP@IOP 0.2 %w.t	H_2_O	r.t	0.5 h	96
16	-	H_2_O	40	24 h	N.R
17	IOP 0.2 %w.t	H_2_O	40	15 min	88

Notes:

^a^ Reaction conditions: 4-nitrobenzaldehyde (3 mmol), *β*-naphthol (3 mmol), and dimedone (3 mmol) and Ag_2_O NP@IOP (0.20 w.t.%, 0.094 g) under different conditions.

Without the presence of the catalyst, the reaction did not perform even after 24 h (Table 1, entry 17). Also, the use of iron pellets alone as a catalyst was less effective and resulted to product formation in a longer time with lower yield (Table 1, entry 18). Nanoparticles due to the high energy of the surfaces tend to agglomerate and become unstable structures. A solution for chemical modification of the surface, economic saving and optimal use of nanocatalyst is the use of supporting substrates and composite synthesis. Placing nanoparticles on the supporting substrate increases the catalyst surface, distribution of active sites, high activity and selectivity, catalyst stability (long life), thermal stability, and decrease the environmental effects.

Finally, in order to find the optimal reaction temperature, the reaction model was investigated at 40°C and ambient temperature. Accordingly, the 40°C was identified as the most effective temperature for the reaction efficiency (Table 1, entry 14), because it took 30 min for the reaction to accomplish at ambient temperature while no significant difference in reaction time and efficiency was observed between the 40°C and the reflux conditions.

Results of optimizing the three factors (*i.e.*, solvent type, catalyst dosage, and reaction temperature) showed that the shortest reaction time coupled with the highest efficiency can be expected with water as solvent in presence of the catalyst at 20 wt% at 40°C (Table 1, entry 14).

In order to synthesize the target compounds, 15 mL of water and Ag_2_O NP@IOP at 20 wt% were added into a mixture of different aromatic benzaldehydes (3 mmol), *β*-naphthol (3 mmol), and dimedone (3 mmol) in a flask. At a temperature of 40°C, the mixture was stirred by a magnetic stirrer. The reaction progress and completion were monitored by TLC. Results are presented in Table 2. Following the completion of the reaction, the produced precipitates were filtered. Then, in order to separate and recover the catalyst, the produced precipitate was dissolved in acetone and a magnet was devised to collect the catalyst particles. All of the obtained compounds were characterized by investigating their melting points and IR spectrometry. To further verify the results, the compound of 12-(2-hydroxy-3-methoxyphenyl)-9,9-dimethyl-8,9,10,12-tetrahydro-11H-benzo[a]xanthen-11-one (4a) was characterized by ^1^H NMR and ^13^C NMR spectroscopy.

**TABLE 2:** Preparation of tetrahydrobenzo[α]xanthen-11-one derivatives in the presence of Ag_2_O NP@IOP nano-catalyst.

Entry^a^	R (aldehyde)	Product	Time (min)	Yield (%)^b^	$\frac{m.p.\;\left(℃\right)}{Found\;Reported\;\left[ref.\right]}$
1	2-OH-3-OCH_3_C_6_H_3_-	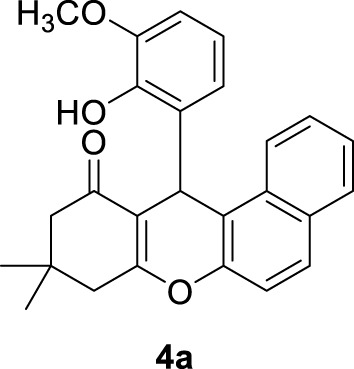	2	94	211–212 213–215 (Fatahpour et al., 2018)
2	2-OH-1-naphthaldehyde	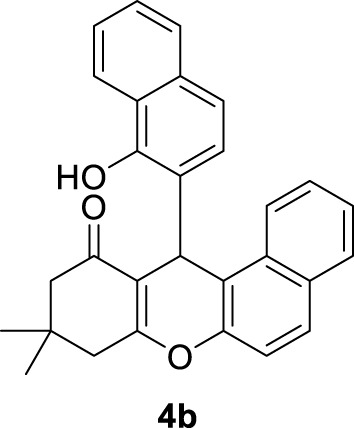	12	99	260–263 New
3	5-Br-2-OHC_6_H_3_-	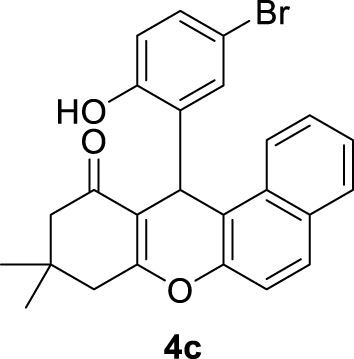	10	98	248–250 248–252 (Fatahpour et al., 2018)
4	4-CH_3_C_6_H_4_-	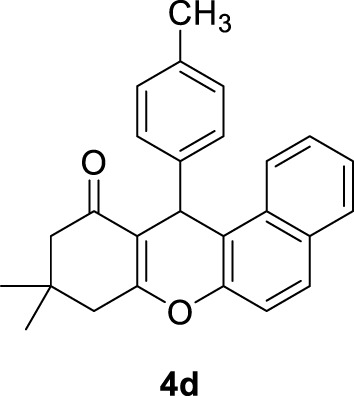	4	95	176–177 176–177 (Fatahpour et al., 2018)
5	2-OCH_3_C_6_H_4_-	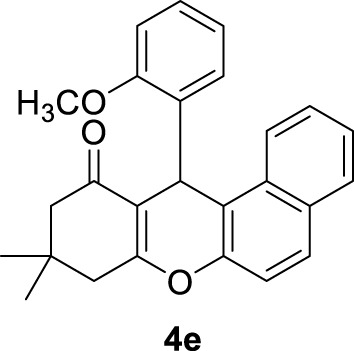	3	98	168–169 166–167 (Kheirkhah et al., 2018)
6	4-OCH_3_C_6_H_4_-	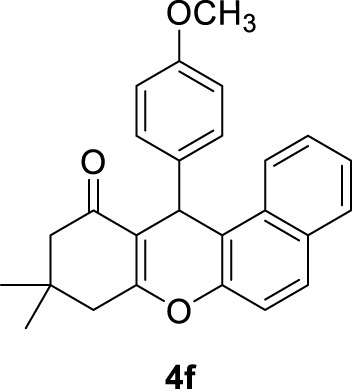	3	98	204–207 203–205 (Saied et al., 2022)
7	4-N(CH_3_)_2_C_6_H_4_-	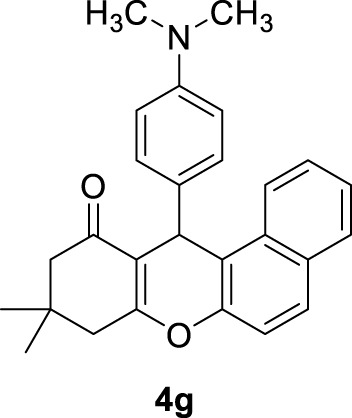	2	99	195–197 196–198 (Sethukumar et al., 2011)
8	4-NO_2_C_6_H_4_-	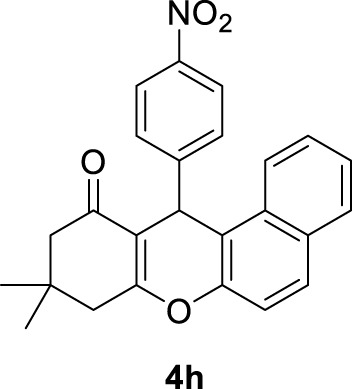	2	90	175–177 176–178 (Saied et al., 2022)
9	3-NO_2_C_6_H_4_-	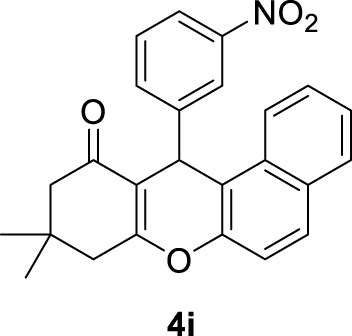	3	97	169–173 170–171 (Shaterian and Mohammadnia, 2013)
10	2,4-(OCH_3_)_2_C_6_H_3_-	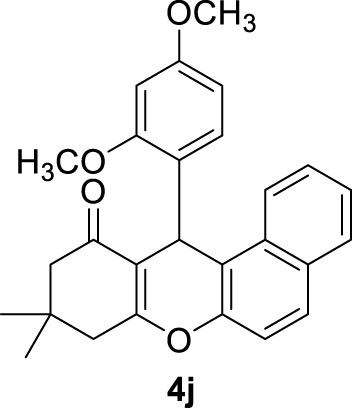	2	98	192–195 New
11	3,4,5-(OCH_3_)_3_C_6_H_2_-	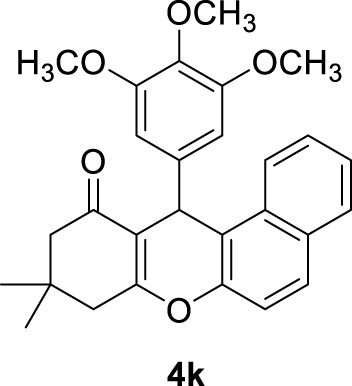	2	99	198–201 197–198 (Esfahani et al., 2013)
12	4-OH-3-OCH_3_C_6_H_3_-	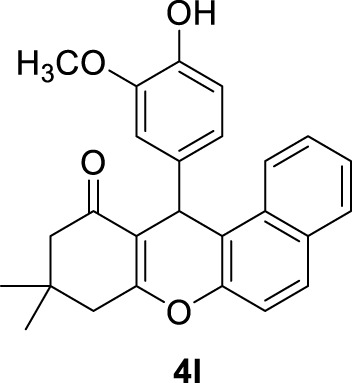	2	98	194–197 196–198 (Agrwal et al., 2021)

Notes:

^a^ Reaction conditions: Aldehyde (3 mmol), *β*-naphthol (3 mmol), dimedone (3 mmol) in the presence of Ag_2_O NP@IOP (20 w.t.%) in H_2_O at temperature of 40 °C.

^b^ Isolated yields after purification.

**SCHEME 4:**
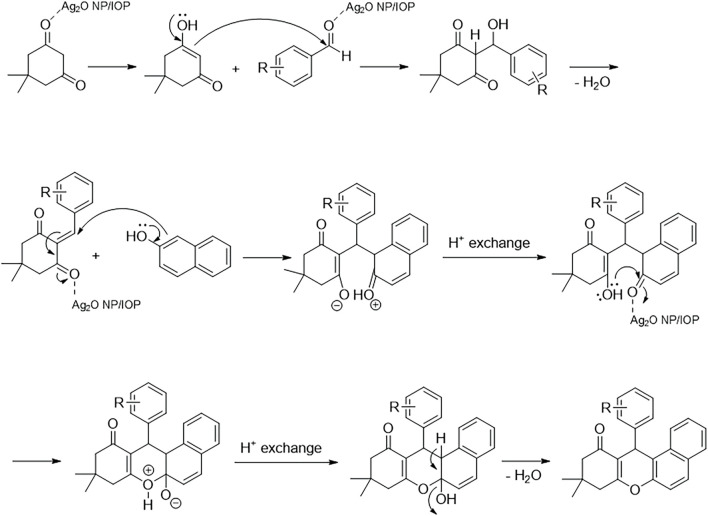
Proposed mechanism for the synthesis of tetrahydrobenzo[α]xanthen-11-one derivatives.

First, as a Lewis acid, the Ag_2_O NP@IOP nanocatalyst established strong coordinate bonds to activate the carbonyl groups on the aldehyde and dimedone, thereby facilitating the enolic nucleophilic attack of the dimedone on the carbonyl group of the aldehyde, which increases the formation rate of the Knoevenagel intermediate. Also, in the next step, the catalyst strengthens Michael conjugate addition of *β*-naphthol from the alpha position, which has a higher electron density, and increases the rate of formation of open chain intermediates. Finally, the intermediate becomes intramolecularly cyclized by the nucleophilic attack of the oxygen electron pair of the hydroxyl dimedone group on the carbonyl carbon of *β*-naphthol, which has been increased by the catalyst, and after a proton exchange step and a water loss step produces the final product (Scheme 4) (Bamoniri et al., 2022; Pirouzmand et al., 2017).

**FIGURE 8:**
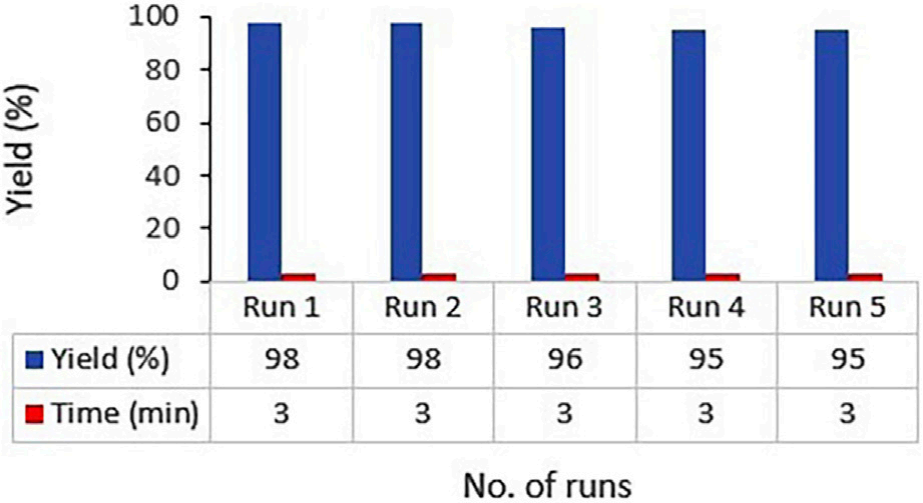
Ag_2_O NP/IOP catalyst recovery diagram in the preparation of tetrahydrobenzo[α]xanthen-11-one derivatives.

Recyclability of catalysts, especially magnetized ones, not only can reduce the expenses related to industrial consumption of the catalyst but also is important in terms of its environmental aspects. Accordingly, we investigated the recyclability and reusability of the heterogenous Ag_2_O NP@IOP catalyst in the reaction model of 4-nitrobenzaldehyde, *β*-naphthol, and dimedone under optimal process conditions for synthesizing the **4h** compound. To this end, following the completion of the product synthesis, the obtained precipitates were filtered. Then, in order to separate and recover the catalyst, the produced precipitate was dissolved in acetone and a magnet was devised to collect the catalyst particles easily. The collected catalyst was several times washed with water and ethanol before being reused in a next run of reaction. Finally, it was figured out that the catalyst can be easily reused for at least three times without losing its catalytic activity. Following an overflow and evaporation of the solvent, the target product was achieved, which could be purified by washing with pure water for only once (Figure 8).

### 3.3 Characterization of Ag_2_O NP@IOP catalyst after recycled procedure

#### 3.3.1 Morphology and particle sized distribution

Further characterization of the recovered Ag_2_O NP@IOP nanocatalyst was done by investigate of FT-IR, SEM, EDX, and X-ray analyses.

**FIGURE 9:**
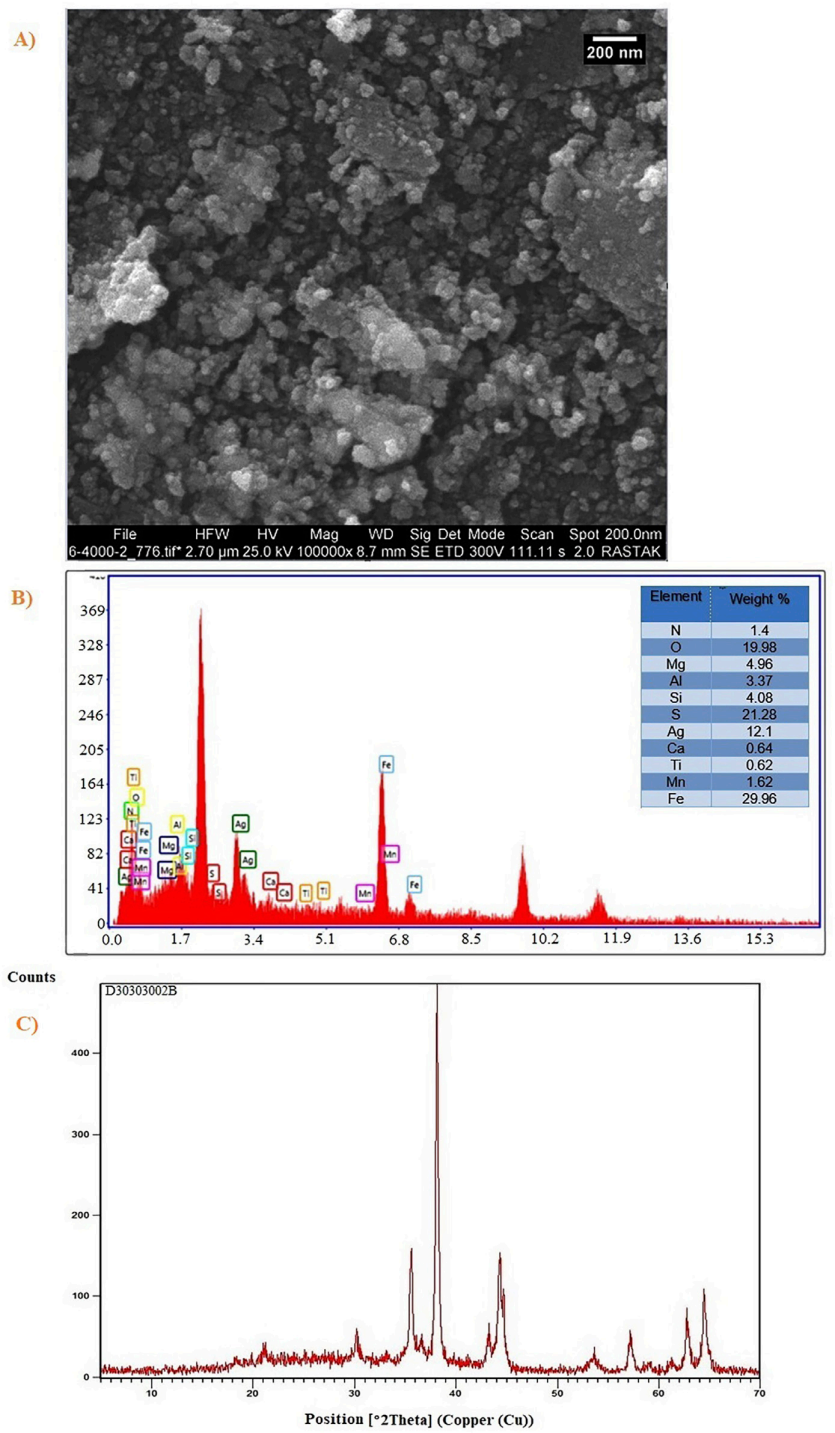
**(A)** SEM image, **(B)** EDAX elemental analysis, and **(C)** XRD patterns of Ag_2_O NP@IOP nanocatalyst after recycled procedure.

Following the recovery procedure, the shape and particle size distribution of the Ag_2_O NP@IOP catalyst were examined using SEM images. After the recovery process, the sample’s morphology tends to resemble a spherical, as seen in Figure 9A. Additionally, the catalytic impacts of Ag_2_O NP@IOP products cause the agreed particles to be seen on the sample’s surface. As can be seen from this figure, the partial instability of the products following the catalytic process is the reason why the average particle size distribution of Ag_2_O NP@IOP has risen.

Figure 9B shows the EDAX elemental analysis of Ag_2_O NP@IOP following the catalytic procedure. The constituents associated with the current Ag_2_O NP@IOP nanocatalyst are clearly visible in the finished product, according to the data obtained. This shows that the final structure of the Ag_2_O NP@IOP nanocatalyst is stable and that the sample is not disturbed after the catalytic process.

Figure 9C displayed the Ag_2_O NP@IOP nanocatalyst’s XRD patterns following the recycling procedure. According to the data obtained, the recycled structure has a good index of the Ag_2_O NP@IOP nanocatalyst’s characterization peaks. The presence of cubic nanocrystals during recycling is further confirmed by the characterization patterns. Further characterization of the Ag_2_O NP@IOP nanocatalyst’s XRD patterns reveals a number of noises in the final structures, which are brought on by the organic compounds in the recycled catalyst. One significant finding is that the JCPDS card number 00-012-0793 corresponds to the nanocatalyst after recycling, which is matched with the XRD patterns before recycling process.

#### 3.3.2 FT-IR spectrum

**FIGURE 10:**
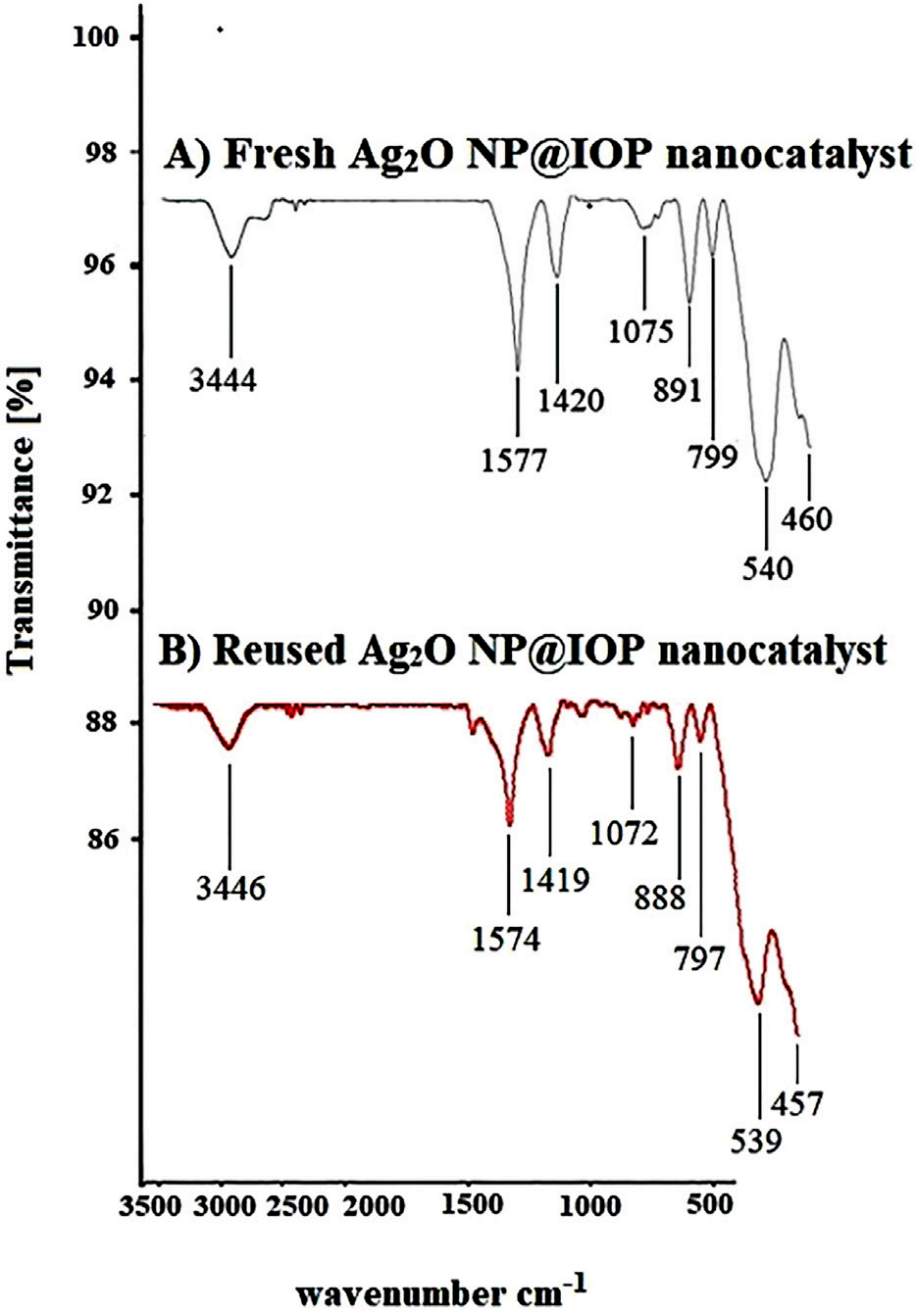
**(A)** FT-IR spectra of fresh Ag_2_O NP@IOP nano-catalyst, and **(B)** FT-IR spectra of Ag_2_O NP@IOP nano-catalyst during the last run (5th) reuse cycle.

When the FT-IR spectra of the recovered catalyst and the fresh catalyst are compared after five runs, a similar pattern is seen, including absorptions of 3,446, 1,574, 1,419, 1,072, 539, and 460 cm^−1^ associated with the iron pellet substrate (IOP) and absorptions of 888 and 797 cm^−1^ associated with the load of silver nanoparticles on the iron pellet and their interactions. This indicates that the structure of the catalyst remains unchanged after recycling and can be reused without structural changes (Figure 10).

#### 3.3.3 Leaching test

The leaching test under optimized reaction conditions was investigated in this the model reaction to better assess the catalyst’s performance. By employing an external magnetic field to separate the Ag_2_O NP@IOP nanocatalysts from the medium after the half-reaction period had passed and the reaction had achieved around 50% completion, a 45% yield was obtained. After then, the filtrate’s progress was let to continue reacting under the same conditions but without a catalyst. Thin-layer chromatography (TLC) examination indicated that no substantial amount of product was obtained after 20 min, the reaction had not advanced, and the substrates had not been converted into the intended product. The heterogeneous nature of the suggested nano-catalyst is further confirmed by these data, which likewise show no leaching and great stability of supported Ag sites.

The Turn-over frequency (TOF) and Turn-over number (TON) values in Table 3 show the superiority of the synthesized Ag_2_O NP@IOP nanocatalyst with other two catalysts in the synthesis process of tetrahydrobenzo[a]xanthen-11-ones through the three-component reaction of dimedone, *β*-naphthol and benzaldehydes. A greater yield and less catalyst use are indicated by larger TON and TOF numerical values, and the catalyst becomes more effective as the values rise.

**TABLE 3:** The comparison of Turn-over frequency (TOF) and Turn-over number (TON) values Ag_2_O NP@IOP nanocatalyst with other two catalysts in the synthesis process of tetrahydrobenzo[α]xanthen-11-one derivatives.

Entry^a^	Catalyst	Amount of catalyst	Conditions	Time (min)	Yield^b^ (%)	TON^c^	TOF (min^−1^) ^d^	Ref
1	GO-SB-H_2_PMo	5.1 mol%	Solvent free, 120°C	5 min	90	17.65	3.53 min^−1^	(Bosica et al., 2023)
2	MWCNTs-SO_3_H	15.5 mol%	Solvent free, 90°C	26 min	92	35.66	1.37 min^−1^	Tashakkorian et al. (2015)
3	Ag_2_O NP@IOP	20 wt%	H_2_O, 40°C	3 min	98	32.143	10.714 min^−1^	This work

^a^ The reaction conditions for the model reaction (1 mmol 4-nitrobenzaldehyde, 1 mmol dimedone and 1 mmol *β*-naphtol): 20 w.t.% catalyst,15 mL H_2_O, 40 °C, 3 min.

^b^ Isolated yield.

^c^ Turnover number (TON) = the mmol of desired product/the mmol of active site of catalyst.

^d^ Turnover frequency [TOF (min−1)] = TON/the reaction time (min).

Using the reaction model of 4-nitrobenzaldehyde, *β*-naphthol, and dimedone in the presence of the green magnetized Ag_2_O NP@IOP catalyst, the results of synthesizing 9,9-dimethyl-12-(4-nitrophenyl)-8,9,10,12-tetrahydro-H11-benzo[a]xanthen-11-one (**4h)** compound were compared to those with other catalysts documented in the literature to demonstrate the efficacy and efficiency of the Ag_2_O NP@IOP catalyst in comparison to other catalysts used for the same product (Table 4). As can be seen from the data in Table 4, the catalyst prepared using the suggested approach in this study is considerably simpler to prepare than many other ways. When the catalyst is magnetized, the reaction time is shortened and the conditions are milder. Numerous techniques for preparing tetrahydrobenzo[α]xanthens from the reaction of benzaldehydes, dimedone, and *β*-naphthol have been reported (Table 4). These techniques have disadvantages, including high temperatures (Table 4, entries 3, 6, 7, 10, 13, 16, 17, 18, 19, and 20), lengthy reaction times (Table 4, entry 15), and hard reaction conditions (Table 4, entries 5, 9, 12). However, in the current study, an Ag_2_O NP@IOP nanocatalyst was created by stabilizing silver nanoparticles on the naturally and magnetic iron pellet (IOP) substrate. Mild reaction conditions, the use of H_2_O as green media and the avoidance of hazardous solvents, clean processes, ease of product isolation, short reaction times, high product yield, reusability and handling of the catalyst, simple catalyst preparation, lower catalytic loading, a green heterogeneous solid acid catalyst as an environmentally friendly catalyst with high catalytic activity, and straightforward experimental and isolation procedures with the use of an external magnet are just a few of the notable benefits that the designed catalyst offers. Additionally, there was no significant loss of catalytic activity for product synthesis when the catalysts were reused up to five times. Therefore, the suggested approach complies with certain green chemistry guidelines and is sustainable, cost-effective and attractive.

**TABLE 4:** Comparison of Ag_2_O NP@IOP nanocatalyst efficiency with a number of catalysts and previous methods presented in the preparation of tetrahydrobenzo[α]xanthen-11-one derivatives.

Entry	Catalyst	Amount of catalyst	Conditions	Time (min/h)	Yield (%)	Ref
1	HAp-encapsulated γ-Fe_2_O_3_ [Fe_2_O_3_@ HAp] -supported dual acidic nanocatalyst	0.02 g	EtOH, 60°C	4 min	96	Kheirkhah et al. (2018)
2	[DSTMG][CF_3_COO]	5 mol%	Solvent free, 75°C	12 min	90	Dutta et al. (2017)
3	[CTA]Fe/MCM-41(DS)	0.1 g	Solvent free, 110°C	25 min	91	Pirouzmand et al. (2017)
4	KO_2_/Et_4_NBr	4 mol%	Dry DMF, r.t	4 h	90	Abel and Ogundana (2014)
5	BF_3_:OEt_2_ and EtOH	0.02 mmol	Reflux	45 min	83	Sethukumar et al. (2011)
6	Citric acid	20 mol%	Solvent free, 120°C	20 min	92	Pawar et al. (2014)
7	IBX	10 mol%	[Hmim]CH_3_SO_3_, 80°C	1 h	92	Chaskar et al. (2011)
8	nano-kaolin-SO_3_H	0.02 g	Solvent free, 70°C	173 min	87	Bamoniri et al. (2022)
9	(Ni_0.5_Co_0.5_Fe_2_O_4_)	20 mol%	EtOH, reflux	30 min	96	Korupolu et al. (2017)
10	lactic acid	50 mol%	Solvent free, 50°C	15 min	88	Fatahpour et al. (2018)
11	[CTA]Fe/MCM-41(DS)	0.1 g	Solvent free, 110°C	25 min	91	Pirouzmand et al. (2017)
12	NiFe_2_O_4_@SiO_2_@ aminoglucose MNPs	0.05 g	solvent-free, r.t	10 min	97	Fekri and Darya-Laal (2019)
13	Fe_3_O_4_@SiO_2_ NPs	0.014 g	EtOH/H_2_O (1:1), reflux	45 min	94	Ghasemzadeh (2015)
14	SrFe_12_O_19_	0.01 g	solvent-free, 80°C	15 min	95	Kheilkordi et al. (2019)
15	γ-Fe_2_O_3_[Fe_2_O_3_@HAp]	20 mol%	EtOH, 60°C	4 min	96	Kheirkhah et al. (2018)
16	Fe_3_O_4_@chitosan	0.03 g	EtOH, 40°C	180 min	90	Maleki et al. (2016)
17	AIL@MN	0.055 g	Neat, 80°C	45 min	89	Zhang et al. (2012)
18	LAIL@MNP	0.015 g	solvent-free sonication, 80°C	30 min	96	Nguyen et al. (2018)
19	Fe_3_O_4_/CS-Ag NPs	0.015 g	H_2_O, 80°C	30 min	94	Mohammadi et al. (2014)
20	Cu (II)/Fe_3_O_4_@ APTMS-DFX a	0.02 g	Solvent free, 120°C	45 min	90	Sonei et al. (2019)
21	Fe_3_O_4_@nano-walnut shell/BIII	0.02 g	Solvent free, 80°C	40 min	95	Abad et al. (2023)
22	Ag_2_O NP@IOP	20 wt%	H_2_O, 40°C	3 min	98	This work

## 4 Conclusion

In conclusion, a new Bi-functional Ag_2_O@IOP nanocatalyst has been introduced as an effective heterogeneous nano-catalyst and also fully characterized by XRD, FE-SEM, EDX, VSM, and BET. The Ag_2_O NP@IOP catalyst was used to prepare heterocyclic derivatives of tetrahydrobenzo[α]xanthen-11-one with important biological and pharmacological effects in water as green condition. High catalytic activity, easy magnetically separation from the reaction mixture and reusability are three significant factors for evaluating the performance of Ag_2_O@IOP nanocatalyst in the organic transformations.

## Data Availability

The original contributions presented in the study are publicly available. This data can be found here: 10.6084/m9.figshare.28594472

## References

[B1] Abad S. S. S. Mirjalili B. B. F. Bamoniri A. (2023). Fe_3_O_4_@ nano-walnut shell/BIII as a new natural based catalyst for synthesis of tetrahydrobenzo [a] xanthene-11-one derivatives. Polycycl. Aromat. Compd. 43, 7979–7991. 10.1080/10406638.2022.2144907

[B2] Abel O. T. Ogundana A. K. (2014). Preliminary quality and potential assessment of groundwater in the basement complex terrain of ijero-ekiti, southwestern Nigeria. Int. J. Innov. Res. Sci. Stud. 3, 15100–15107. 10.15680/IJIRSET.2014.0308007

[B3] Agrwal A. Kumar V. Kasana V. (2021). Preparation and application of highly efficient and reusable TBAPIL@ Si(CH_2_)_3_@ nano-silica-based nano-catalyst for preparation of benzoxanthene derivatives. J. Iran. Chem. Soc. 18, 2583–2595. 10.1007/s13738-021-02211-1

[B4] Bamoniri A. Yaghmaeiyan N. Yaghmaeiyan N. (2022). Synthesis of 9, 9-dimethyl-12-(aryl)-8, 9, 10, 12-tetrahydrobenzo [a] xanthene-11-ones by modified kaolinite nanoclay as an efficient and reusable heterogeneous catalyst via a green protocol. Indian J. Chem. 61, 599–606. 10.56042/ijc.v61i6.64203

[B5] Bedi P. Behera A. K. Alanazi A. K. Roy M. Das S. Shukla R. (2023). Benzoxanthones derivatives: insight into oxalic acid catalyzed synthesis, crystallographic analysis and potential application in dye-sensitized solar cell. Chem. Sci. J. 135, 34–47. 10.1007/s12039-023-02151-8

[B6] Bogen S. T. Karolin J. Molotkovsky J. G. Johansson L. B. A. (1998). 1, 32-Dihydroxy-dotriacontane-bis (Rhodamine) 101 ester A lipid membrane spanning bichromophoric molecule as revealed by intramolecular donor–donor energy migration (DDEM). J. Chem. Soc. Faraday Trans. 94, 2435–2440. 10.1039/A801970C

[B7] Bosica G. De Nittis R. Borg R. (2023). Solvent-free, one-pot, multicomponent synthesis of xanthene derivatives. J. Catal. 13, 561–578. 10.3390/catal13030561

[B8] Bossi M. Belov V. Polyakova S. Hell S. W. (2006). Reversible red fluorescent molecular switches. Angew. Chem. - Int. Ed. 45, 7462–7465. 10.1002/anie.200602591 17042053

[B9] Bossi M. Folling J. Belov V. N. Boyarskiy V. P. Medda R. Egner A. (2008). Multicolor far-field fluorescence nanoscopy through isolated detection of distinct molecular species. Nano Lett. 8, 2463–2468. 10.1021/nl801471d 18642961

[B10] Chaskar A. Shaikh H. Padalkar V. Phatangare K. Deokar H. (2011). IBX promoted one-pot condensation of *β*-naphthol, aldehydes, and 1, 3-dicarbonyl compounds. Green Chem. Lett. Rev. 4, 171–175. 10.1080/17518253.2010.528047

[B11] Collard X. Li L. Lueangchaichaweng W. Bertrand A. Aprile C. Pescarmona P. P. (2014). Ga-MCM-41 nanoparticles: synthesis and application of versatile heterogeneous catalysts. Catal. Today 235, 184–192. 10.1016/j.cattod.2014.02.055

[B12] Crosby G. A. Demas J. N. (1971). Measurement of photoluminescence quantum yields. Review. Rev. Phys. Chem. 75, 991–1024. 10.1021/j100678a001

[B13] Das B. Laxminarayana K. Krishnaiah M. Srinivas Y. (2007). An efficient and convenient protocol for the synthesis of novel 12-aryl-or 12-alkyl-8, 9, 10, 12-tetrahydrobenzo[a]xanthen-11-one derivatives. Synlett 2007, 3107–3112. 10.1055/s-2007-990922

[B14] Dasaradhudu Y. Srinivasan M. A. (2020). Synthesis and characterization of silver nano particles using co-precipitation method. Mater. Today Proc. 33, 720–723. 10.1016/j.matpr.2020.06.029

[B16] Drexhage K. H. (1976). Fluorescence efficiency of laser dyes. J. Res. Natl. Bur. Stand A Phys. Chem. 80, 421–428. 10.6028/jres.080A.044

[B17] Dutta A. K. Gogoi P. Saikia S. Borah R. (2017). N, N-disulfo-1, 1, 3, 3- tetramethylguanidinium carboxylate ionic liquids as reusable homogeneous catalysts for multicomponent synthesis of tetrahydrobenzo [a] xanthene and tetrahydrobenzo [a] acridine derivatives. J. Mol. Liq. 225, 585–591. 10.1016/j.molliq.2016.11.112

[B15] Esfahani H. R. Foroughifar N. Mobinikhaledi A. Moghanian H. (2013). Ammonium oxalate as an efficient catalyst for one-pot synthesis of tetrahydrobenzo [a] xanthen-11-one derivatives under solvent-free conditions. Syn React Inorg Metaorg Nanometal Chem. 43, 752–755. 10.1080/15533174.2012.754767

[B18] Farinha J. P. S. Charreyre M. T. Martinho J. M. G. Winnik M. A. Pichot C. (2001). Picosecond fluorescence studies of the surface morphology of charged polystyrene latex particles. Langmuir 17, 2617–2623. 10.1021/la001338+

[B19] Fatahpour M. Hazeri N. Maghsoodlou M. T. Lashkari M. (2018). Lactic acid: a new application as an efficient catalyst for the green one-pot synthesis of 2-hydroxy-12-aryl-8, 9, 10, 12-tetrahydrobenzo [a] xanthene-11-one and 12-aryl-8, 9, 10, 12- tetrahydrobenzo [a] xanthen-11-one Analogs. Iran. J. Sci. Technol. Trans. A Sci. 42, 533–538. 10.1007/s40995-016-0064-1

[B20] Fekri L. Z. Darya-Laal A. R. (2019). NiFe_2_O_4_@ SiO_2_@ amino glucose magnetic nanoparticle as a green, effective and magnetically separable catalyst for the synthesis of xanthene-ones under solvent-free condition. Polycycl. Aromat. Compd. 40, 1539–1556. 10.1080/10406638.2018.1559207

[B21] Fonseca T. Relogio P. Martinho J. M. G. Farinha J. P. S. (2007). Preparation and surface characterization of polymer nanoparticles designed for incorporation into hybrid materials. Langmuir 23, 5727–5734. 10.1021/la063381o 17417887

[B22] Ghasemipour P. Fattahi M. Rasekh B. Yazdian F. (2020). Developing the ternary ZnO doped MoS_2_ nanostructures grafted on CNT and reduced graphene oxide (RGO) for photocatalytic degradation of aniline. Sci. Rep. 10, 4414. 10.1038/s41598-020-61367-7 32157131 PMC7064525

[B23] Ghasemzadeh A. M. Safaei-Ghomi J. Zahedi S. (2013). Fe_3_O_4_ nanoparticles: a highly efficient and easily reusable catalyst for the one-pot synthesis of xanthene derivatives under solvent-free conditions. J. Serb. Chem. Soc. 78, 769–779. 10.2298/JSC120624156G

[B24] Ghasemzadeh M. A. (2015). Synthesis and characterization of Fe_3_O_4_@ SiO_2_ NPs as an effective catalyst for the synthesis of tetrahydrobenzo [a] xanthen-11-ones. Acta Chim. Slov. 62, 977–985. 10.17344/acsi.2015.1501 26680728

[B25] Haq S. Yasin K. A. Rehman W. Waseem M. Ahmed M. N. Shahzad M. I. (2021). Green synthesis of silver oxide nanostructures and investigation of their synergistic effect with moxifloxacin against selected microorganisms. J. Inorg. Organomet. Polym. Mater. 31, 1134–1142. 10.1007/s10904-020-01763-8

[B26] He L. L. Li X. Y. Bai J. Y. Li S. Qi S. Wang X. (2022). A novel ZnWO_4_/MgWO_4_ nn heterojunction with enhanced sonocatalytic performance for the removal of methylene blue: characterizations and sonocatalytic mechanism. Surf. Interfaces 31, 101980. 10.1016/j.surfin.2022.101980

[B27] Ho D. O. N. Sun X. Sun S. (2011). Monodisperse magnetic nanoparticles for theranostic applications. Acc. Chem. Res. 44, 875–882. 10.1021/ar200090c 21661754 PMC3184307

[B28] Imon M. K. Islam R. Karmaker P. G. Roy P. K. Lee K. I. Roy H. N. (2022). A concise metal-free synthesis of xanthene derivatives mediated by achiral 2-aminophenol under solvent-free conditions. Synth. Commun. 52, 712–723. 10.1080/00397911.2022.2047730

[B29] Jha A. Beal J. (2004). Convenient synthesis of 12H-benzo[a]xanthenes from 2-tetralone. Tetrahedron Lett. 45, 8999–9001. 10.1016/j.tetlet.2004.10.046

[B30] Jung Y. Kim Y. Lee Y. Son J. Lim M. Nam J. M. (2024). Selective flocculation and H_2_O_2_-free oxidative etching-based synthesis of highly monodisperse Ag nanospheres for uniform quantum dot photoluminescence-enhancing plasmonic cavity applications. J. Am. Chem. Soc. 146, 10591–10598. 10.1021/jacs.4c00073 38570931

[B31] Kheilkordi Z. Ziarani G. M. Lashgari N. Badiei A. (2019). An efficient method for the synthesis of functionalized 4H-chromenes as optical sensor for detection of Fe^3+^ in ethanol. Polyhedron 166, 203–209. 10.1016/j.poly.2019.03.042

[B32] Kheirkhah L. Mamaghani M. Yahyazadeh A. Mahmoodi N. O. (2018). HAp‐encapsulated γ‐Fe_2_O_3_‐supported dual acidic heterogeneous catalyst for highly efficient one‐pot synthesis of benzoxanthenones and 3‐pyranylindoles. Appl. Organomet. Chem. 32, e4072. 10.1002/aoc.4072

[B33] Kim H. N. Lee M. H. Kim H. J. Kim J. S. Yoon J. (2008). A new trend in rhodamine-based chemosensors: application of spirolactam ring-opening to sensing ions. Chem. Soc. Rev. 37, 1465–1472. 10.1039/B802497A 18648672

[B34] Korupolu R. B. Maripi S. Madasu S. B. Majji R. K. Ganta R. K. Chilla P. N. (2017). Nano nickel-cobalt ferrite catalyzed one pot synthesis of 14-Aryl-14H-dibenzo [a, j] xanthenes and 12-Aryl-8, 9, 10, 12-tetrahydrobenzo [a] xanthene-11-one derivatives. Orient. J. Chem. 33, 122–133. 10.13005/ojc/330113

[B35] Kowada T. Maeda H. Kikuchi K. (2015). BODIPY-based probes for the fluorescence imaging of biomolecules in living cells. Chem. Soc. Rev. 44, 4953–4972. 10.1039/C5CS00030K 25801415

[B36] Li L. Tian X. Zou G. Shi Z. Zhang X. Jin W. (2008). Quantitative counting of single fluorescent molecules by combined electrochemical adsorption accumulation and total internal reflection fluorescence microscopy. J. Anal. Chem. 80, 3999–4006. 10.1021/ac702534h 18442261

[B37] Liu W. Howarth M. Greytak A. B. Zheng Y. Nocera D. G. Ting A. Y. (2008). Compact biocompatible quantum dots functionalized for cellular imaging. J. Am. Chem. Soc. 130, 1274–1284. 10.1021/ja076069p 18177042 PMC2665712

[B38] Liu Y. C. Liu X. Wang X. Li Z. H. Chen C. L. Xiang Z. (2023). Hybrid persulfate/sonocatalysis for degradation of acid orange 7 in the presence of Ag_2_O/CuWO_4_ composite: operating parameters and sonocatalytic mechanism. J. Clean. Prod. 394, 136287. 10.1016/j.jclepro.2023.136287

[B39] Ma Z. Linnenberg O. Rokicinska A. Kustrowski P. Slabon A. (2018). Augmenting the photocurrent of CuWO_4_ photoanodes by heat treatment in the nitrogen atmosphere. J. Phys. Chem. 122, 19281–19288. 10.1021/acs.jpcc.8b02828

[B40] Madhav J. V. Kuarm B. S. Rajitha B. (2008). Dipyridine cobalt chloride: a novel and efficient catalyst for the synthesis of 14-aryl-14H-dibenzo[a, j]xanthenes under solvent-free conditions. Arkivoc 2, 204–209. 10.3998/ark.5550190.0009.222

[B41] Maleki A. Aghaei M. Ghamari N. (2016). Facile synthesis of tetrahydrobenzoxanthenones via a one‐pot three‐component reaction using an eco‐friendly and magnetized biopolymer chitosan‐based heterogeneous nanocatalyst. Appl. Organomet. Chem. 30, 939–942. 10.1002/aoc.3524

[B42] Ma’mani L. Sheykhan M. Heydari A. Faraji M. Yamini Y. (2010). Sulfonic acid supported on hydroxyapatite-encapsulated-γ-Fe_2_O_3_ nanocrystallites as a magnetically Bronsted acid for N-formylation of amines. Appl. Catal. A Gen. 377, 64–69. 10.1016/j.apcata.2010.01.020

[B43] Mohammadi R. Eidi E. Ghavami M. Kassaee M. Z. (2014). Chitosan synergistically enhanced by successive Fe_3_O_4_ and silver nanoparticles as a novel green catalyst in one-pot, three-component synthesis of tetrahydrobenzo [α] xanthene-11-ones. J. Mol. Catal. A Chem. 393, 309–316. 10.1016/j.molcata.2014.06.005

[B44] Nguyen D. T. Salek S. Shultz‐Johnson L. R. Belanger‐Bouliga M. Jurca T. Byers J. C. (2024). Poly (N‐heterocyclic carbene) ‐capped alloy and core‐shell AuAg bimetallic nanoparticles. Angew. Chem. 136, e202409800. 10.1002/anie.202409800 38887177

[B45] Nguyen H. T. Le N. P. T. Chau D. K. N. Tran P. H. (2018). New nano-Fe_3_O_4_-supported Lewis acidic ionic liquid as a highly effective and recyclable catalyst for the preparation of benzoxanthenes and pyrroles under solvent-free sonication. RSC Adv. 8, 35681–35688. 10.1039/C8RA04893B 35547886 PMC9087937

[B46] Nicolas J. San Miguel V. Mantovani G. Haddleton D. M. (2006). Fluorescently tagged polymer bioconjugates from protein derived macroinitiators. Chem. Commun. 45, 4697–4699. 10.1039/B609935A 17109040

[B47] Patel A. R. Maity G. Pati T. K. Adak L. Cioffi C. L. Banerjee S. (2024). Hybrid Pd_0.1_Cu_0_._9_Co_2_O_4_ nano-flakes: a novel, efficient and reusable catalyst for the one-pot heck and Suzuki couplings with simultaneous transesterification reactions under microwave irradiation. Front. Chem. 12, 1496234. 10.3389/fchem.2024.1496234 39539394 PMC11557397

[B48] Pawar P. B. Jadhav S. D. Deshmukh M. B. Patil S. (2014). Citric acid as a mild and inexpensive organocatalyst for synthesis of tetrahydrobenzo [α] xanthen-11-ones and dibenzo [a, j] xanthenes under solvent-free condition. Indian J. Chem. 53B, 1185–1193.

[B49] Pirouzmand M. Gharehbaba A. M. Ghasemi Z. Khaaje S. A. (2017). [CTA] Fe/MCM-41: an efficient and reusable catalyst for green synthesis of xanthene derivatives. Arab. J. Chem. 10, 1070–1076. 10.1016/j.arabjc.2016.06.017

[B50] Prazeres T. J. Fedorov A. Barbosa S. P. Martinho J. M. Berberan-Santos M. N. (2008). Accurate determination of the limiting anisotropy of rhodamine 101. Implications for its use as a fluorescence polarization standard. J. Phys. Chem. A 112, 5034–5039. 10.1021/jp710625j 18476678

[B51] Prazeres T. J. V. Fedorov A. Martinho J. M. G. (2004a). Dynamics of oligonucleotides adsorbed on thermosensitive core−shell latex particles. J. Phys. Chem. B 108, 9032–9041. 10.1021/jp0489931

[B52] Prazeres T. J. V. Santos A. M. Martinho J. M. G. Elaïssari A. Pichot C. (2004b). Adsorption of oligonucleotides on PMMA/PNIPAM core− shell latexes: polarity of the PNIPAM shell probed by fluorescence. Langmuir 20, 6834–6840. 10.1021/la049609u 15274592

[B53] Quitevis E. L. Marcus A. H. Fayer M. D. (1993). Dynamics of ionic lipophilic probes in micelles: picosecond fluorescence depolarization measurements. J. Phys. Chem. 97, 5762–5769. 10.1021/j100123a049

[B54] Saied S. M. Saleh M. Y. Hamdoon A. M. (2022). Multicomponent synthesis of tetrahydrobenzo[a]xanthene and tetrahydrobenzo[a]acridine derivatives using sulfonated multi-walled carbon nanotubes as heterogeneous nanocatalysts. Iran. J. Catal. 12, 189–205. 10.30495/IJC.2022.1955651.1924

[B55] Schlick T. L. Ding Z. Kovacs E. W. Francis M. B. (2005). Dual-surface modification of the tobacco mosaic virus. J. Am. Chem. Soc. 127, 3718–3723. 10.1021/ja046239n 15771505

[B56] Sethukumar A. Chandy M. M. Arul Prakasam B. Pallepogu R. (2011). Synthesis and spectral studies on some tetrahydrobenzoxanthen-11-ones: crystal and molecular structure of 9, 9-dimethyl-12-(2-nitrophenyl)-8, 9, 10, 12-tetrahydrobenzo [a] xanthen-11-one. J. Struct. Chem. 22, 671–680. 10.1007/s11224-011-9737-8

[B57] Shaterian H. R. Mohammadnia M. (2013). Nanocrystalline TiO_2_–HClO_4_ catalyzed three-component preparation of derivatives of 1-amidoalkyl-2-naphthol, 1- carbamato-alkyl-2-naphthol, 1-(α-aminoalkyl)-2-naphthol, and 12-aryl-8, 9, 10, 12- tetrahydrobenzo [α]-xanthen-11-one. Res. Chem. Intermed. 39, 4221–4237. 10.1007/s11164-012-0938-6

[B58] Sheikhhosseini E. Sattaei M. T. Faryabi M. Rafiepour A. Soltaninejad S. (2016). Iron ore pellet, a natural and reusable catalyst for synthesis of pyrano [2, 3-d] pyrimidine and dihydropyrano [c] chromene derivatives in aqueous media. Iran. J. Chem. Chem. Eng. 35, 43–50.

[B59] Shiraishi Y. Miyamoto R. Hirai T. (2008). Rhodamine-conjugated acrylamide polymers exhibiting selective fluorescence enhancement at specific temperature ranges. J. Photochem. Photobiol. A Chem. 200, 432–437. 10.1016/j.jphotochem.2008.08.020

[B60] Shiraishi Y. Miyamoto R. Zhang X. Hirai T. (2007). Rhodamine-based fluorescent thermometer exhibiting selective emission enhancement at a specific temperature range. Org. Lett. 9, 3921–3924. 10.1021/ol701542m 17727287

[B61] Shirini F. Abedini M. Akbari-Dadamahaleh S. Rahmaninia A. (2014a). Iranian chemist’s efforts to provide various effective methods for the synthesis of xanthenes. J. Iran. Chem. Soc. 11, 791–824. 10.1007/s13738-013-0353-y

[B62] Shirini F. Yahyazadeh A. Mohammadi K. (2014b). One-pot synthesis of various xanthene derivatives using ionic liquid 1, 3-disulfonic acid imidazolium hydrogen sulfate as an efficient and reusable catalyst under solvent-free conditions. Chin. Chem. Lett. 25, 341–347. 10.1016/j.cclet.2013.11.016

[B63] Sonei S. Gholizadeh M. Taghavi F. (2019). Cu (II) anchored on modified magnetic nanoparticles: as a green and efficient recyclable nano catalyst for one pot synthesis of 12-Aryl-8, 9, 10, 12tetrahydrobenzo [a] xanthene-11-one. Polycycl. Aromat. Compd. 40, 1127–1142. 10.1080/10406638.2018.1531431

[B64] Sullivan K. T. Wu C. Piekiel N. W. Gaskell K. Zachariah M. R. (2013). Synthesis and reactivity of nano-Ag_2_O as an oxidizer for energetic systems yielding antimicrobial products. Combust. Flame 160, 438–446. 10.1016/j.combustflame.2012.09.011

[B65] Tashakkorian H. Lakouraj M. M. Rouhi M. (2015). *p*-Sulfonic acid calix [4] arene as an efficient catalyst for one-pot synthesis of pharmaceutically significant coumarin derivatives under solvent-free condition. Int. J. Med. Chem. 2015, 1–8. 10.1155/2015/738202 PMC4698933 26798517

[B66] Veljovic E. Harej A. Klobucar M. Kraljevic Pavelic S. Spirtovic-Halilovic S. Osmanovic A. (2022). Antimikrobno i antiproliferativno djelovanje derivata ksanten-3-ona i njihov afinitet vezivanja za enzime. Kem. Ind. 71, 131–140. 10.15255/KUI.2021.030

[B67] Wang X. Yu S. Li Z. H. He L. L. Liu Q. L. Hu M. Y. (2021). Fabrication Z-scheme heterojunction of Ag_2_O/ZnWO_4_ with enhanced sonocatalytic performances for meloxicam decomposition: increasing adsorption and generation of reactive species. Chem. Eng. J. 405, 126922. 10.1016/j.cej.2020.126922

[B68] Warsi A. Z. Hussien O. K. Iftikhar A. Aziz F. Alhashmialameer D. Mahmoud S. F. (2022). Co-precipitation assisted preparation of Ag_2_O, CuO and Ag_2_O/CuO nanocomposite: characterization and improved solar irradiated degradation of colored and colourless organic effluents. Ceram. Int. 48, 19056–19067. 10.1016/j.ceramint.2022.03.194

[B69] Whitaker J. E. Haugland R. P. Prendergast F. G. (1991). Spectral and photophysical studies of benzo [c] xanthene dyes: dual emission pH sensors. Anal. Biochem. 194, 330–344. 10.1016/0003-2697(91)90237-N 1862936

[B70] Xie G. Leo K. (2022). Catalyzing n-doping. J. Innov. 3, 100219. 10.1016/j.xinn.2022.100219 PMC8904609 35280230

[B71] Xu L. Liu N. P. An H. L. Ju W. T. Liu B. Wang X. F. (2022). Preparation of Ag_3_PO_4_/CoWO_4_ S-scheme heterojunction and study on sonocatalytic degradation of tetracycline. Ultrason. Sonochem. 89, 106147. 10.1016/j.ultsonch.2022.106147 36087545 PMC9465027

[B72] Yu L. Lin C. Liao C. Zeng X. Chen X. Zhu Z. (2020). Green chemistry: efficient acetalization of aldehydes with alcohols using the acid red 52 photocatalyst. Environ. Chem. Lett. 18, 1353–1359. 10.1007/s10311-020-00994-y

[B73] Zhang L. Dang Y. Zhou X. Gao P. van Bavel A. P. Wang H. (2021). Direct conversion of CO_2_ to a jet fuel over CoFe alloy catalysts. J. Innov. 2, 100170. 10.1016/j.xinn.2021.100170 PMC8523875 34704085

[B74] Zhang Q. Su H. Luo J. Wei Y. (2012). A magnetic nanoparticle supported dual acidic ionic liquid: a “quasi-homogeneous” catalyst for the one-pot synthesis of benzoxanthenes. Green Chem. 14, 201–208. 10.1039/C1GC16031A

