# Supplementary material for: Iron ore pellets based-Ag2O nanoparticles as efficient Bi-functional heterogeneous catalyst for the synthesis tetrahydrobenzo[α]xanthens in green media
Source: Front Chem. 2025 Mar 19;13:1413080. doi: 10.3389/fchem.2025.1413080 (PMC11962902; doi:10.3389/fchem.2025.1413080)
Supplement: Supplementary file 1 [file DataSheet1.pdf]

Supplementary Material

Iron Ore pellets based-Ag_2O nanoparticles as efficient Bi-functional heterogeneous catalyst for the synthesis tetrahydrobenzo[α]xanthens in green media

Ehsan Faryabi [1], Enayatollah Sheikhhosseini [1]*, Mahdieh Yahyazadehfar [1]

E-mail: sheikhhosseiny@gmail.com or sheikhhosseini@iauk.ac.ir

This file has been converted from its original format for security purposes. Please use C138DD0810875 as a reference.

IR, ^{1}H NMR and ^{13}C NMR of compound (4a)

***12-(2-hydroxy-3-methoxyphenyl)-9,9-dimethyl-8,9,10,12-tetrahydro-11H-benzo[a]xanthen-11-one (4a)*:** Yield: 94%. M.p. = 211-212 °C. ^{1}H NMR (250 MHz, $CDCl_3$): δ = 0.98 (s, 3H, CH_3), 1.10 (s, 3H, CH_3), 1.96 (brs, 2H, CH_2), 2.33 (d, *J*= 5.75 Hz, 1H, CH_2), 2.58 (d, *J*= 10 Hz, 1H, CH_2), 3.85 (s, 3H, OCH_3), 4.67 (s, 1H, CH), 6.57-6.91 (m, 7H, H-Ar), 7.27-7.36 (m, 1H, H-Ar), 7.73-7.80 (m, 1H, H-Ar), 10.41 (brs, 1H, OH). ^{13}C NMR (62.5 MHz, $CDCl_3$): δ = 26.2, 26.8, 38.6, 40.2, 47.0, 47.6, 53.1, 107.4, 107.94, 113. 7, 115.2, 116.8, 117.8, 120.7, 121.5, 122.3, 124.4, 125.2, 126.0, 137.6, 137.9, 144.1, 144.5, 165.7, 167.8, 198.3.

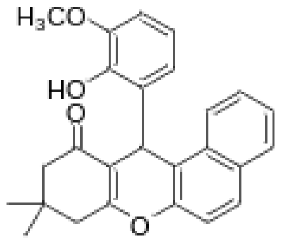

4a

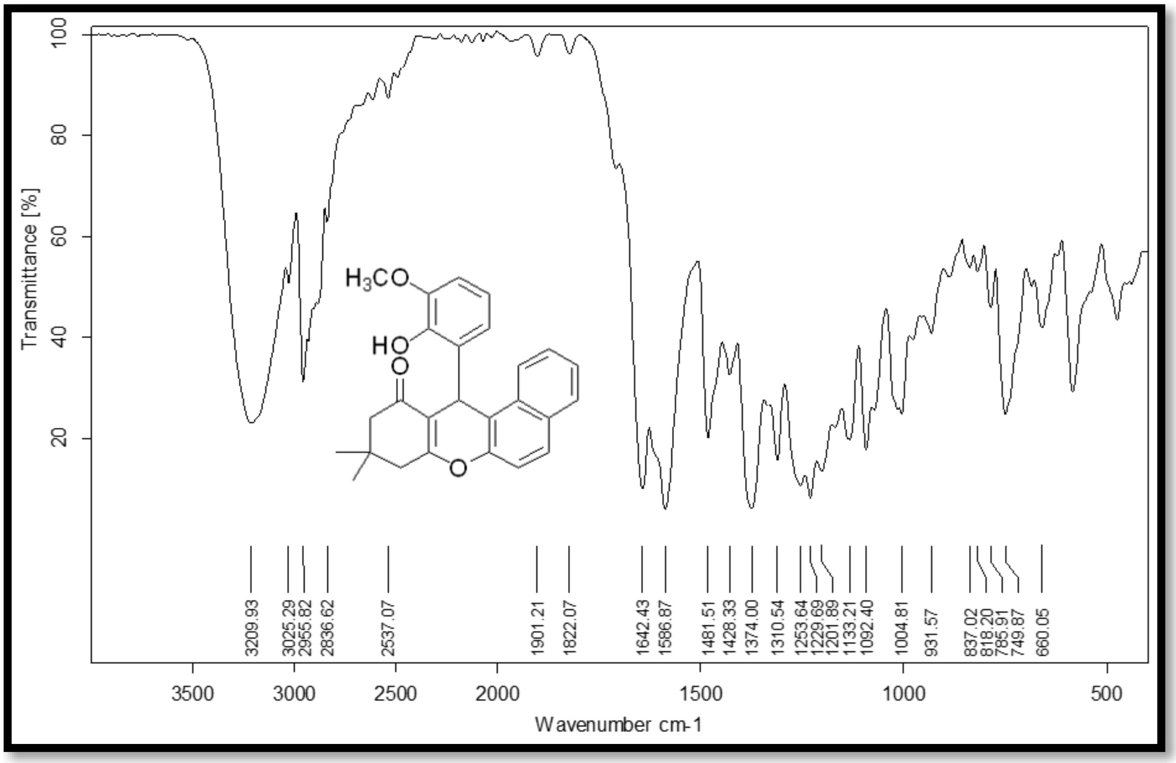

Fig. 1. FT-IR spectrum of compound 4a.

This file has been converted from its original format for security purposes. Please use C138DD0810875 as a reference.

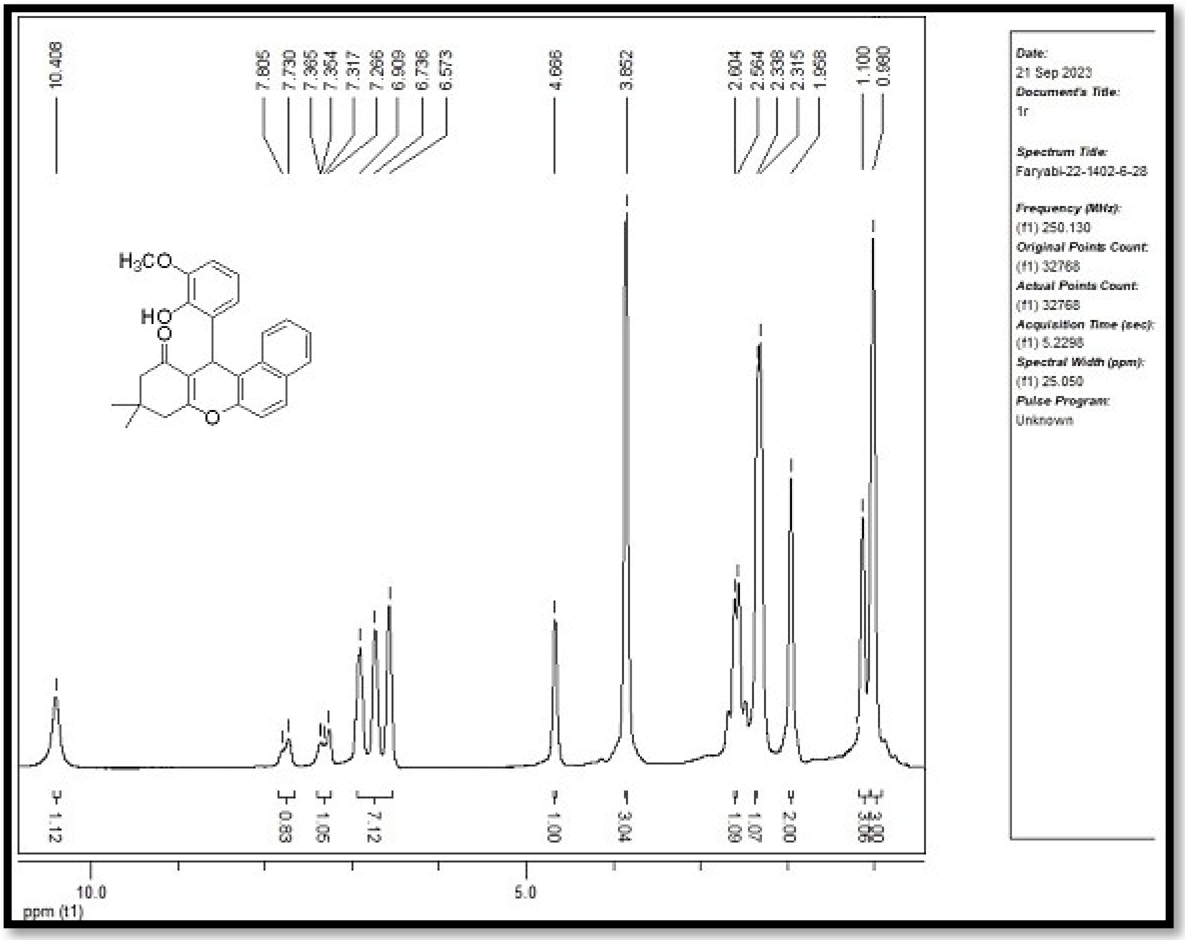

Fig. 2. ^{1}H NMR spectrum of compound 4a.

This file has been converted from its original format for security purposes. Please use C138DD0810875 as a reference.

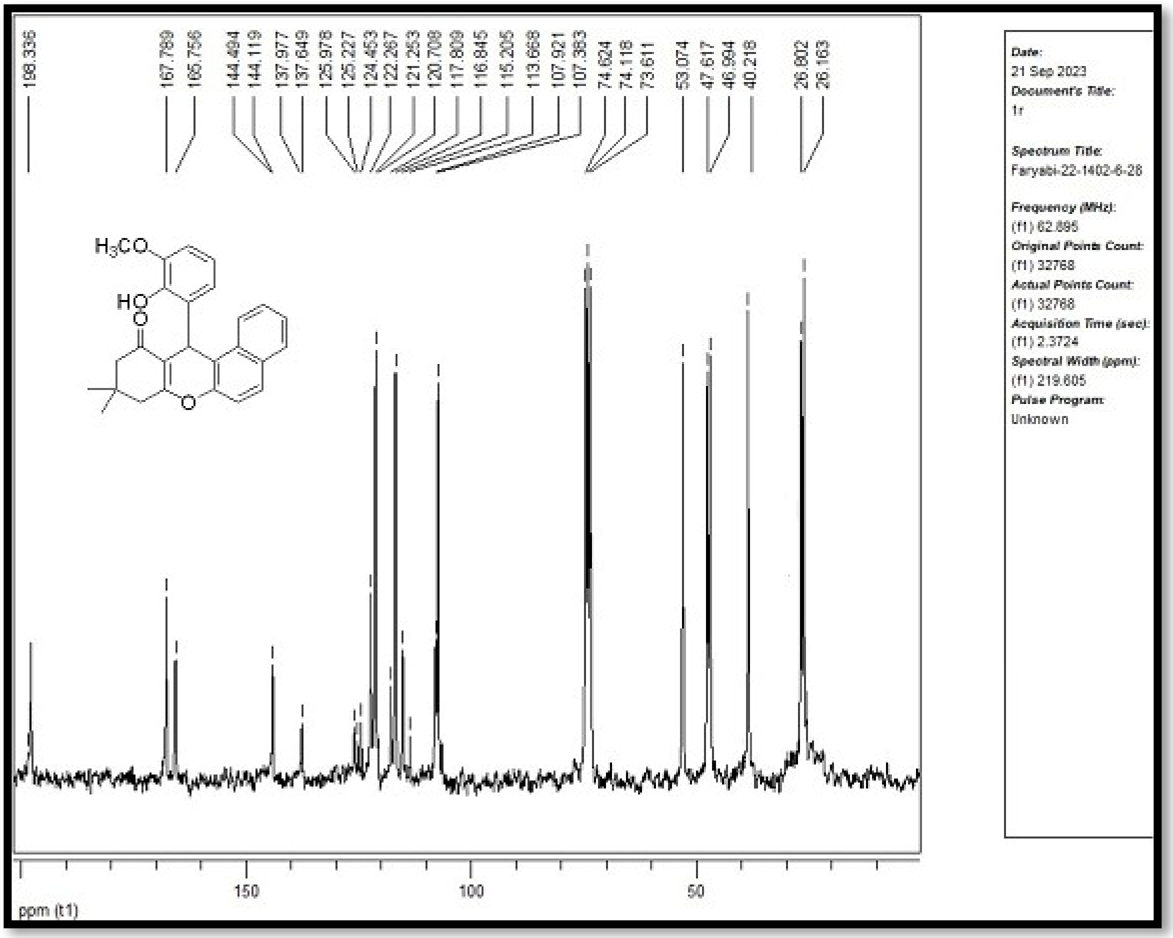

Fig. 3. ^{1}C NMR spectrum of compound 4a.

This file has been converted from its original format for security purposes. Please use C138DD0810875 as a reference.

IR, ^{1}H NMR and ^{13}C NMR of compound (4b)

12-(3-hydroxynaphthalen-2-yl)-9,9-dimethyl-8,9,10,12-tetrahydro-11H-benzo[a]xanthen-11-one (4b): Yield: 99%. M,p. = 260-263 °C. IR (KBr, cm^{-1}): 3175, 2946, 2892, 2863, 1644, 1594, 1373, 1011-1333, 791-980. ^{1}H NMR (250 MHz, $CDCl_3$): δ = 1.05 (s, 3H, CH_3), 1.15 (s, 3H, CH_3), 1.88 (d, *J*= 18 Hz, 1H, CH_2), 2.39 (brs, 2H, CH_2), 2.60 (d, *J*= 9.25 Hz, 1H, CH_2), 5.25 (s, 1H, CH), 7.26-7.45 (m, 6H, H-Ar), 7.72 (brs, 6H, H-Ar), 10.68 (brs, 1H, OH). ^{13}C NMR (62.5 MHz, $CDCl_3$): δ = 26.4, 26.9, 38.4, 40.2, 47.0, 47.8, 108.1, 112.8, 113.2, 113.6, 114.4, 114.7, 119.9, 120.6, 121.7, 122.5, 123.0, 123.2, 123.7, 124.4, 125.5, 126.4, 128.0, 128.3, 129.0, 145.9, 166.1, 167.3, 198.1.

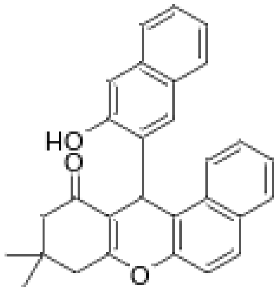

4b

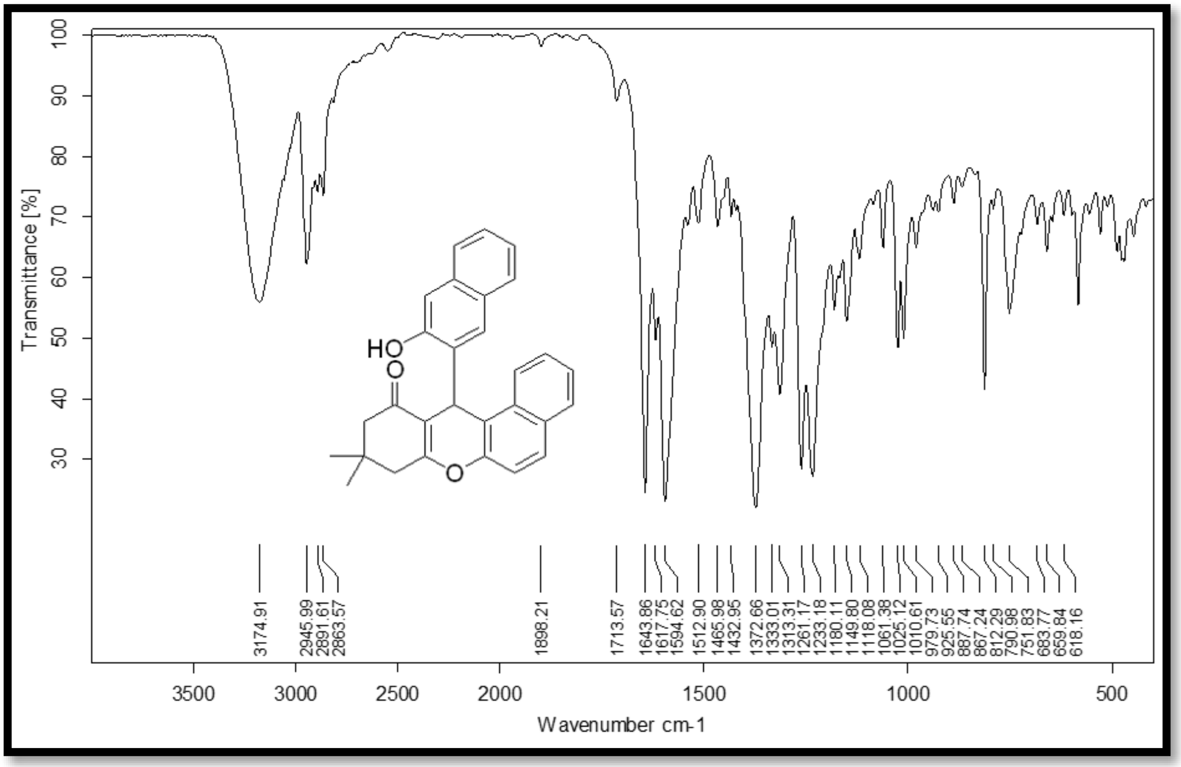

Fig. 4. FT-IR spectrum of compound 4b.

This file has been converted from its original format for security purposes. Please use C138DD0810875 as a reference.

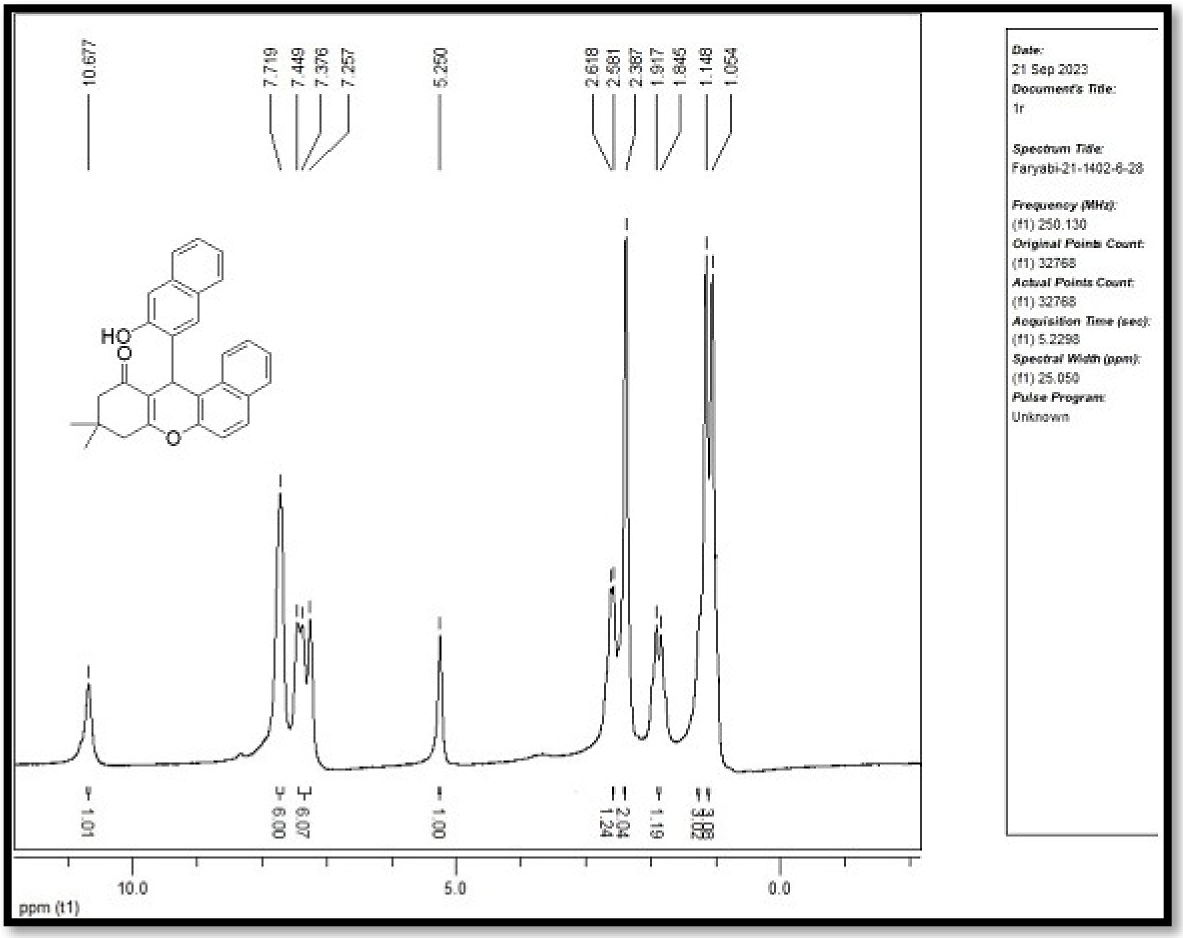

Fig. 5. ^{1}H NMR spectrum of compound 4b.

This file has been converted from its original format for security purposes. Please use C138DD0810875 as a reference.

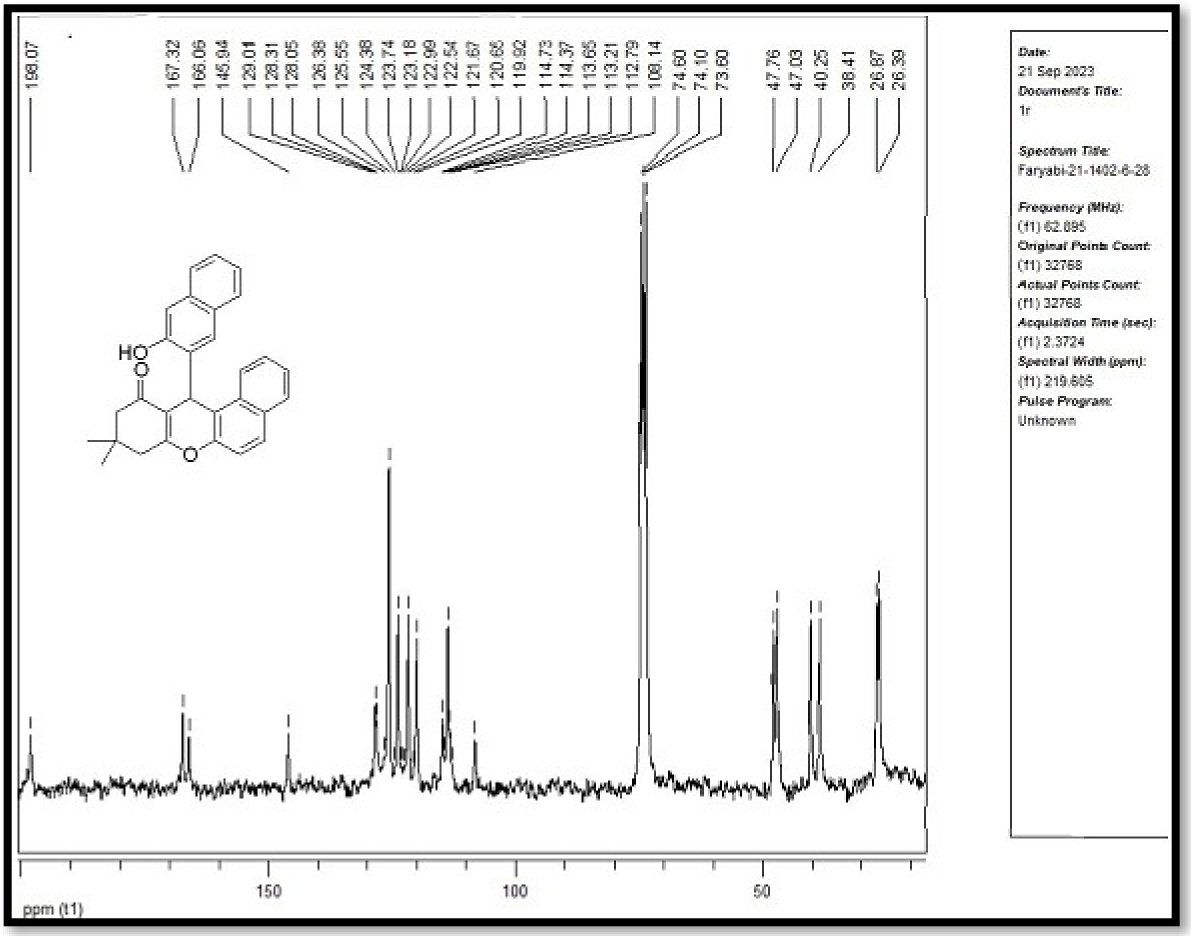

Fig. 6. ^{13}C NMR spectrum of compound 4b.

This file has been converted from its original format for security purposes. Please use C138DD0810875 as a reference.

^{1}H NMR of compound (4c)

12-(5-bromo-2-hydroxyphenyl)-9,9-dimethyl-8,9,10,12-tetrahydro-11H-benzo[a]xanthen-11-one (4c): Yield: 98%. M,p. = 248-250 °C. ^{1}H NMR (DMSO-d_6, 250 MHz): δ = 0.91 (s, 6H, 2 CH_3), 2.06-2.47 (m, 4H, 2CH_2), 5.01 (s, 1H, CH), 6.62-6.65 (m, 1H, H-Ar), 6.99-7.40 (m, 5H, H-Ar), 7.83-7.85 (m, 2H, H-Ar), 8.21-8.24 (m, 1H, H-Ar), 9.99 (brs, 1H, OH).

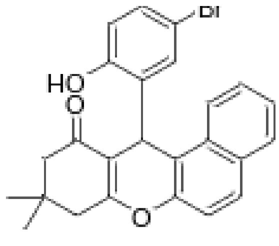

4c

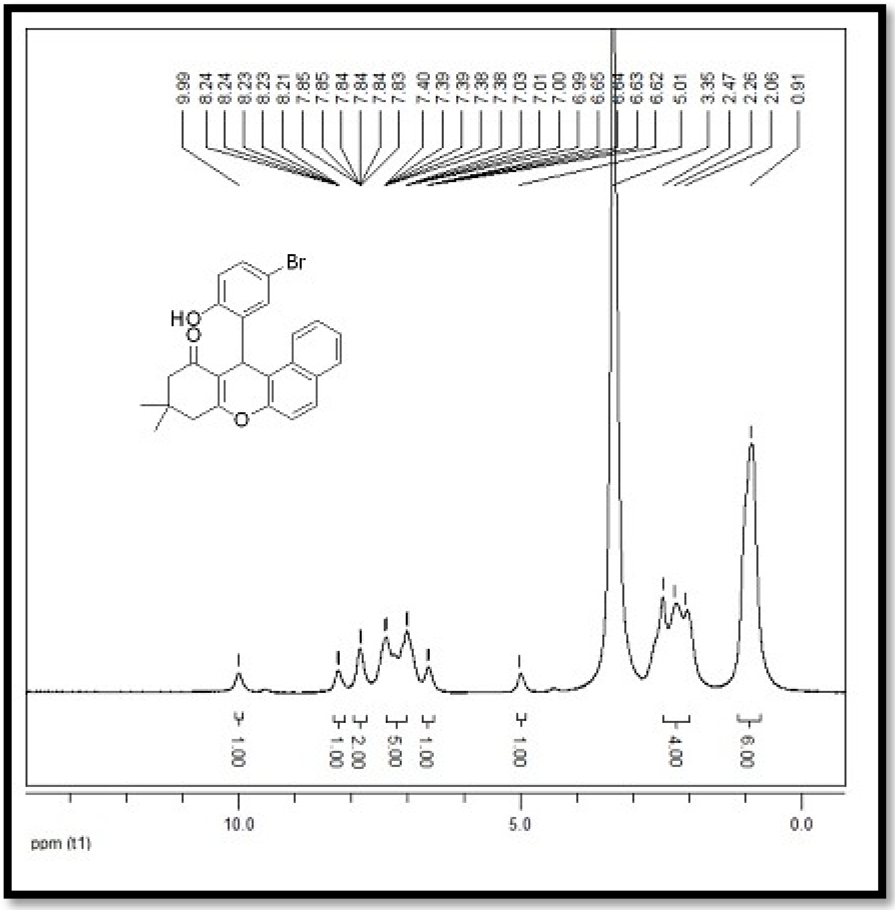

Fig. 7. ^{1}H NMR spectrum of compound 4c.

This file has been converted from its original format for security purposes. Please use C138DD0810875 as a reference.

^{1}H NMR of compound (4d)

9,9-dimethyl-12-(p-tolyl)-8,9,10,12-tetrahydro-11H-benzo[a]xanthen-11-one (4d): Yield: 95%. M,p. = 176-177 $^{\circ}$C. IR (KBr, cm^{-1}): 3175, 2946, 2892, 2863, 1644, 1594, 1373, 1011-1333, 791-980. ^{1}H NMR (250 MHz, DMSO-d_6): δ = 0.85 (s, 3H, CH_3), 1.02 (s, 3H, CH_3), 2.10 (s, 3H, CH_3), 2.27-2.57 (m, 4H, $2CH_2$), 5.49 (s, 1H, CH), 6.95-7.98 (m, 10H, H-Ar).

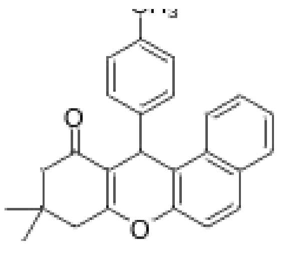

4d

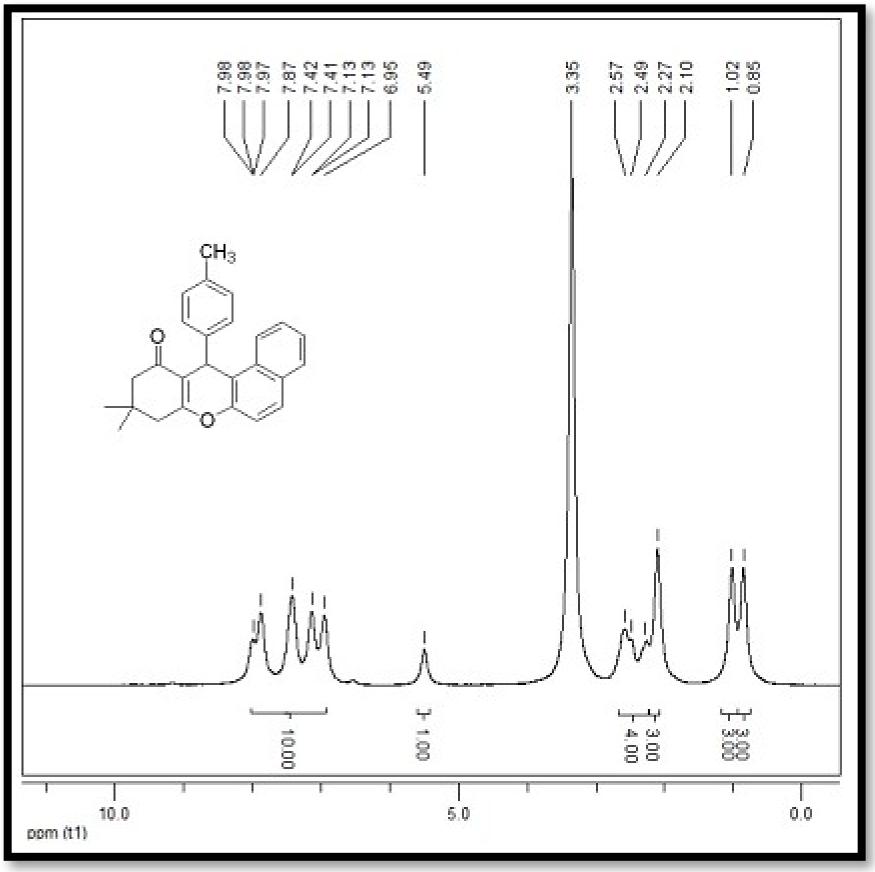

This file has been converted from its original format for security purposes. Please use C138DD0810875 as a reference.

Fig. 8. ^{1}H NMR spectrum of compound 4d.

^{1}H NMR of compound (4e)

12-(2-methoxyphenyl)-9,9-dimethyl-8,9,10,12-tetrahydro-11H-benzo[a]xanthen-11-one (4e): Yield: 98%. M,p. = 168-169 ˚C. ^{1}H NMR (250 MHz, DMSO-d_6): δ = 0.82 (s, 3H, CH_3), 1.07 (s, 3H, CH_3), 2.02 (d, *J*= 20 Hz, 2H, CH_2), 2.21 (brs, 2H, CH_2), 3.70 (s, 3H, OCH_3), 5.81 (s, 1H, CH), 6.67-7.03 (m, 10 H, H-Ar).

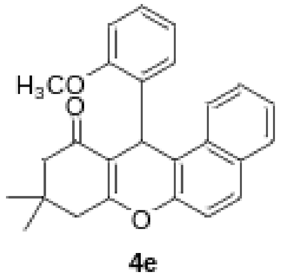

4e

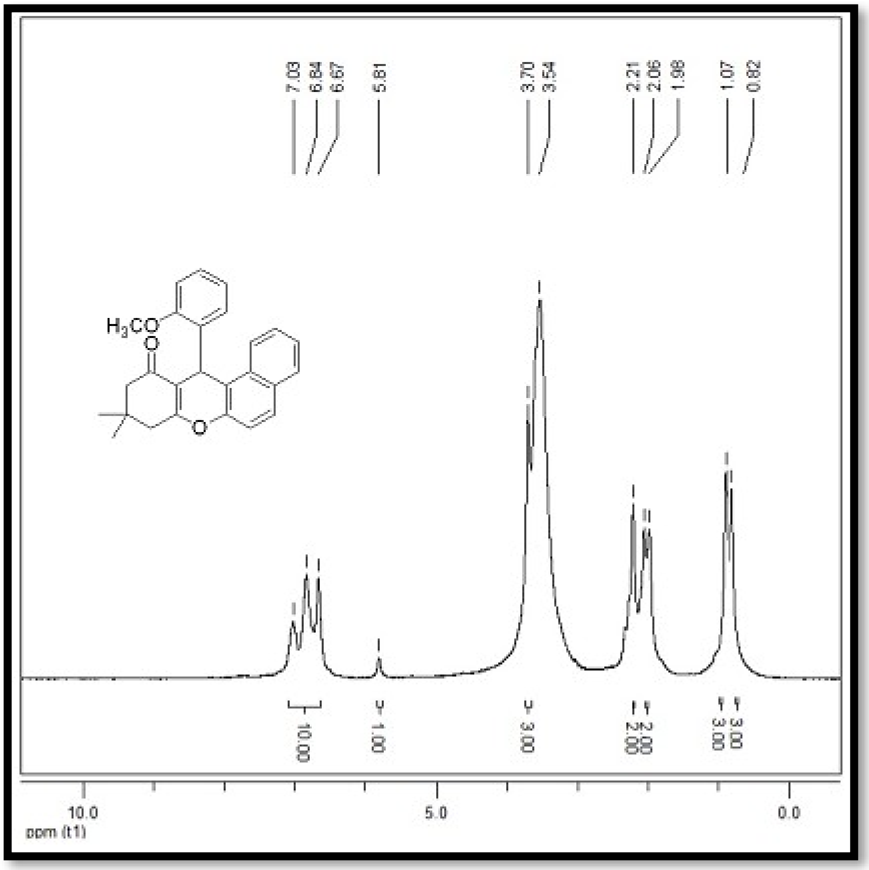

Fig. 9. ^{1}H NMR spectrum of compound 4e.

This file has been converted from its original format for security purposes. Please use C138DD0810875 as a reference.

^{1}H NMR of compound (4f)

12-(4-methoxyphenyl)-9,9-dimethyl-8,9,10,12-tetrahydro-11H-benzo[a]xanthen-11-one ***(4f)*****:** Yield: 98%. M,p. = 204-207 °C. ^{1}H NMR (250 MHz, DMSO-d_6): δ = 0.86 (s, 3H, CH_3), 0.99 (s, 3H, CH_3), 2.05-2.48 (m, 4H, $2CH_2$), 3.64 (s, 3H, OCH_3), 4.41 (s, 1H, CH), 6.73-7.04 (m, 10 H, H-Ar).

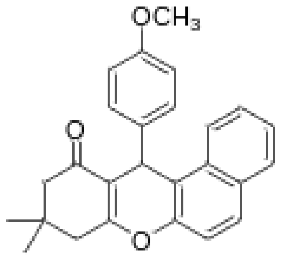

4f

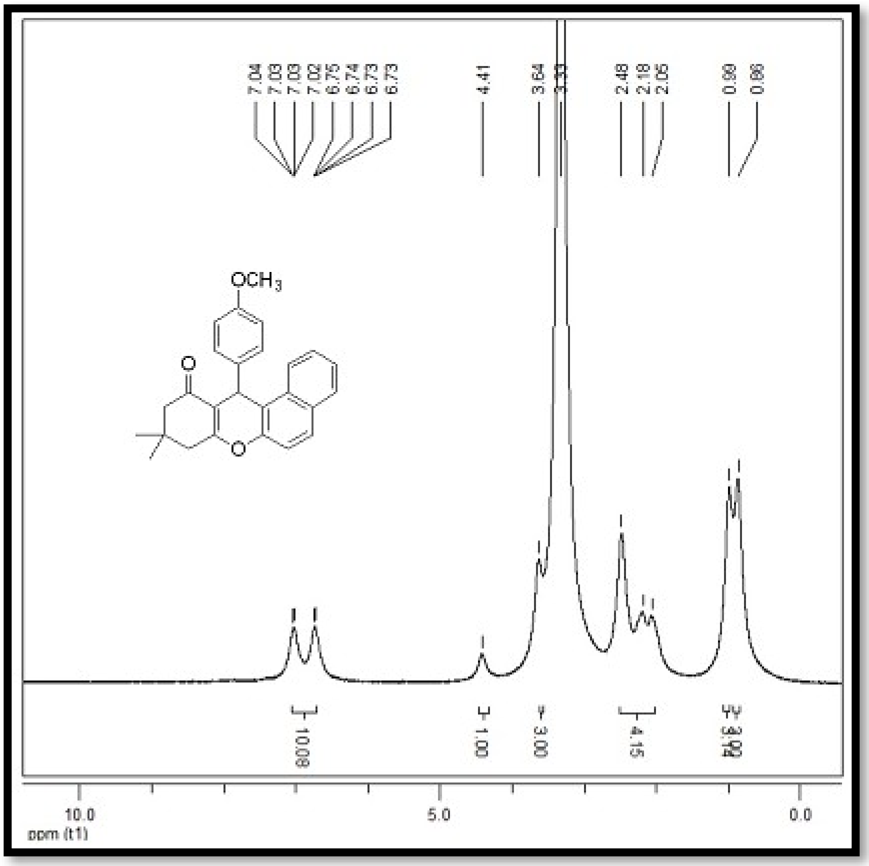

Fig. 10. ^{1}H NMR spectrum of compound 4f.

This file has been converted from its original format for security purposes. Please use C138DD0810875 as a reference.

^{1}H NMR of compound (4g)

12-(4-(dimethylamino)phenyl)-9,9-dimethyl-8,9,10,12-tetrahydro-11H-benzo[a]xanthen-11-one (4g): Yield: 99%. M,p. = 195-197 ˚C. ^{1}H NMR (250 MHz, DMSO-d_6): δ = 1.01 (s, 6H, 2CH_3), 2.29 (brs, 2H, CH_2), 2.79 (brs, 2H, CH_2), 3.26 (s, 6H, 2CH_3), 5.73 (s, 1H, CH), 6.55-6.76 (m, 10 H, H-Ar).

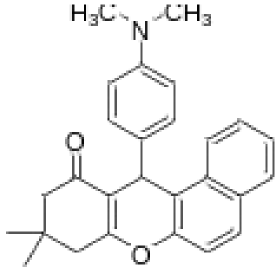

4g

This file has been converted from its original format for security purposes. Please use C138DD0810875 as a reference.

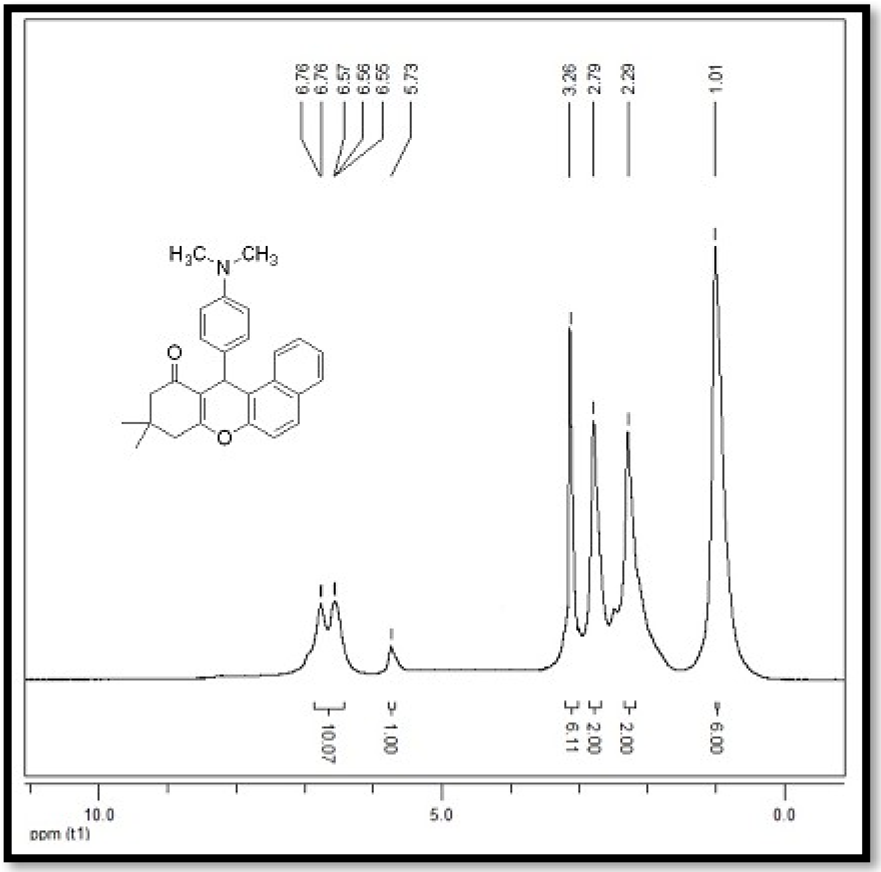

Fig. 11. ^{1}H NMR spectrum of compound 4g.

^{1}H NMR of compound (4h)

***9,9-dimethyl-12-(4-nitrophenyl)-8,9,10,12-tetrahydro-11H-benzo[a]xanthen-11-one (4h)*:** Yield: 90%. M,p. = 175-177 ˚C. ^{1}H NMR (250 MHz, DMSO-d_6): δ = 0.83 (s, 3H, CH_3), 1.01 (s, 3H, CH_3), 2.06 (d, *J*= 20 Hz, 2H, CH_2), 2.32 (brs, 2H, CH_2), 5.73 (s, 1H, CH), 7.18 (d, *J*= 5 Hz, 2H, H-Ar), 7.32 (d, *J*= 5 Hz, 2H, H-Ar), 7.41 (d, *J*= 7.5 Hz, 2H, H-Ar), 7.57 (d, *J*= 7.5 Hz, 2H, H-Ar), 8.04 (t, *J*= 7.5 Hz, 2H, H-Ar).

This file has been converted from its original format for security purposes. Please use C138DD0810875 as a reference.

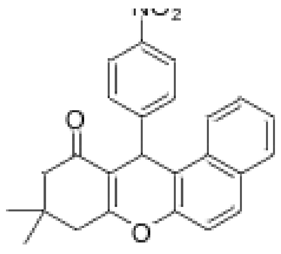

4h

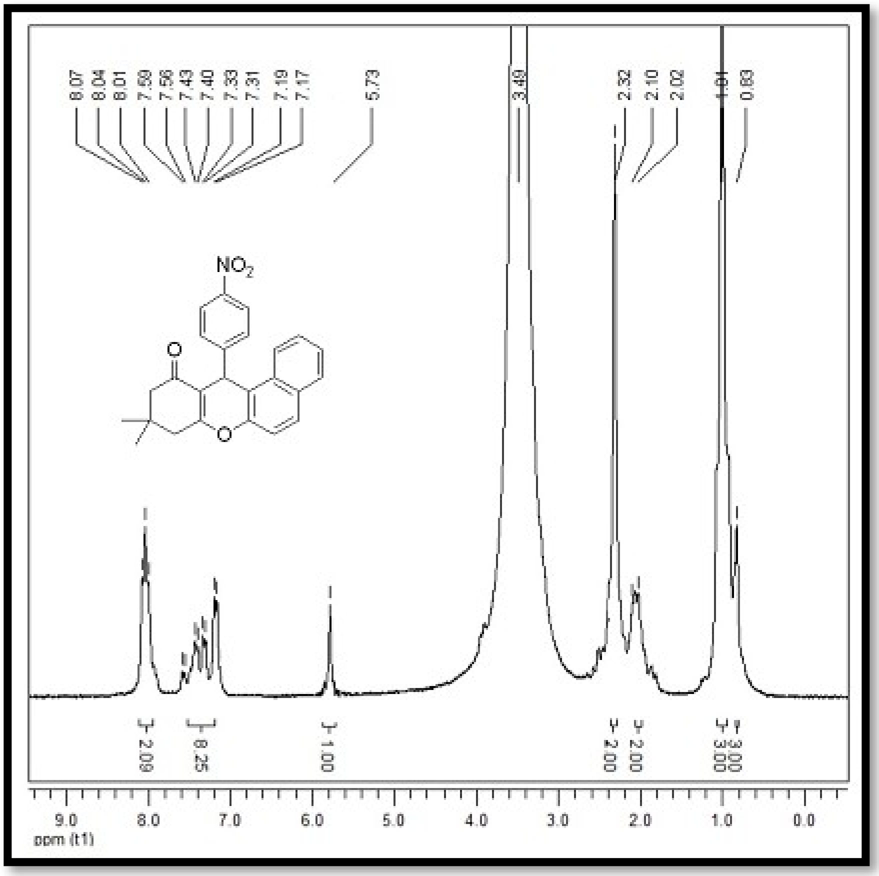

Fig. 12. ^{1}H NMR spectrum of compound 4h.

^{1}H NMR of compound (4i)

***9,9-dimethyl-12-(3-nitrophenyl)-8,9,10,12-tetrahydro-11H-benzo[a]xanthen-11-one (4i)*:** Yield: 97%. M,p. = 169-173 ˚C. ^{1}H NMR (250 MHz, DMSO-d_6): δ = 1.02 (s, 6H, 2CH_3), 2.06-2.46 (m, 4H, 2CH_2), 5.77 (s, 1H, CH), 6.99-7.08 (m, 2H, H-Ar), 7.47-7.94 (m, 8H, H-Ar).

This file has been converted from its original format for security purposes. Please use C138DD0810875 as a reference.

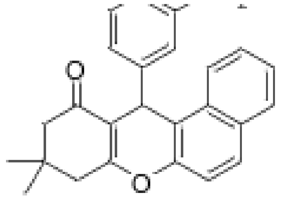

4i

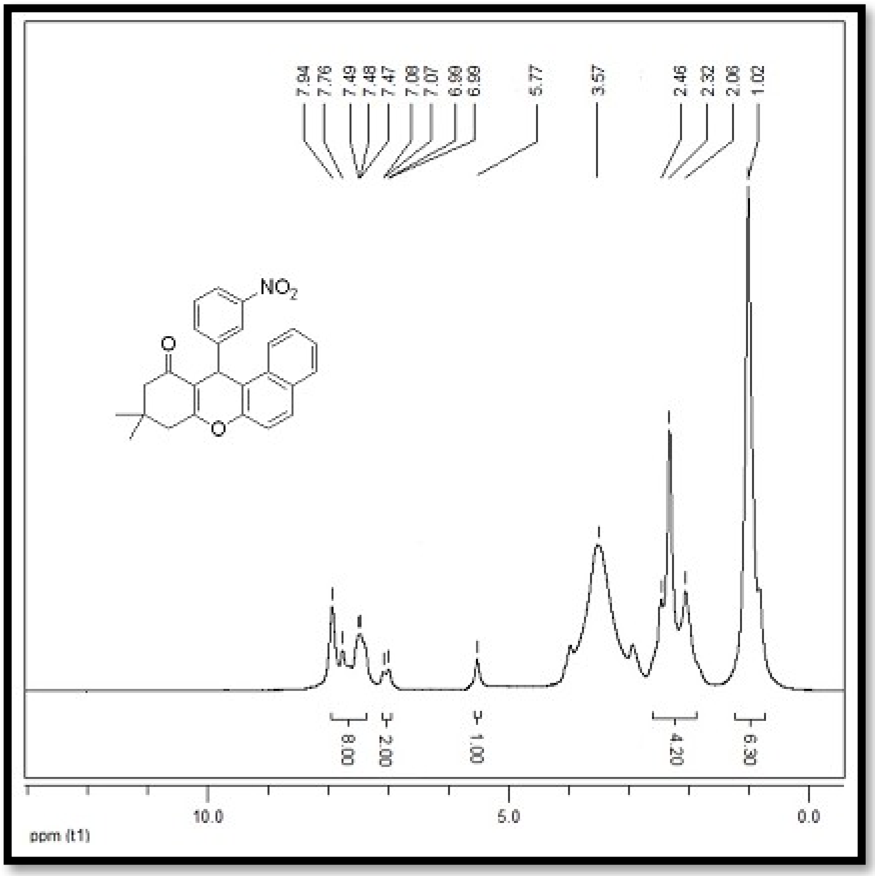

Fig. 13. ^{1}H NMR spectrum of compound 4i.

^{1}H NMR of compound (4j)

***12-(2,4-dimethoxyphenyl)-9,9-dimethyl-8,9,10,12-tetrahydro-11H-benzo[a]xanthen-11-one (4j)*:** Yield: 98%. M,p. = 192-195 ˚C. ^{1}H NMR (250 MHz, DMSO-d_6): δ = 0.95 (s, 6H, $2CH_3$), 2.01-2.47 (m, 4H, $2CH_2$), 3.88 (s, 6H, $2OCH_3$), 5.69 (s, 1H, CH), 6.27-6.72 (m, 9H, H-Ar).

This file has been converted from its original format for security purposes. Please use C138DD0810875 as a reference.

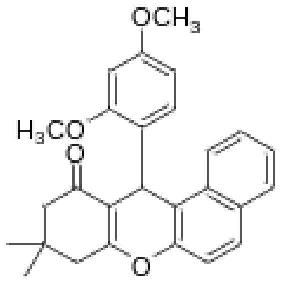

4j

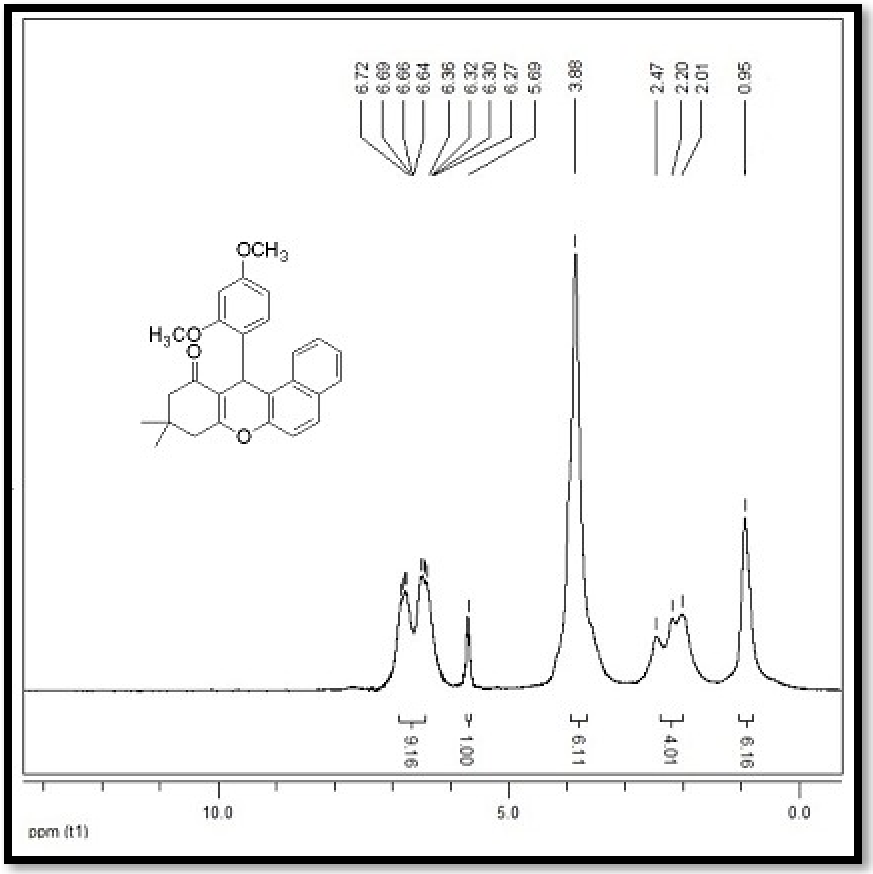

Fig. 14. ^{1}H NMR spectrum of compound 4j.

^{1}H NMR of compound (4k)

***9,9-dimethyl-12-(3,4,5-trimethoxyphenyl)-8,9,10,12-tetrahydro-11H-benzo[a]xanthen-11-one (4k)*:** Yield: 99%. M,p. = 198-201 ˚C. ^{1}H NMR (250 MHz, DMSO-d_6): δ = 1.03 (s, 6H, 2CH_3), 2.06-2.48

This file has been converted from its original format for security purposes. Please use C138DD0810875 as a reference.

(m, 4H, 2CH_2), 3.62 (s, 9H, 3OCH_3), 5.82 (s, 1H, CH), 6.22-6.41 (m, 6H, H-Ar), 6.76 (brs, 1H, H-Ar), 6.86-6.87 (m, 1H, H-Ar).

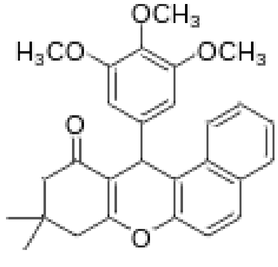

4k

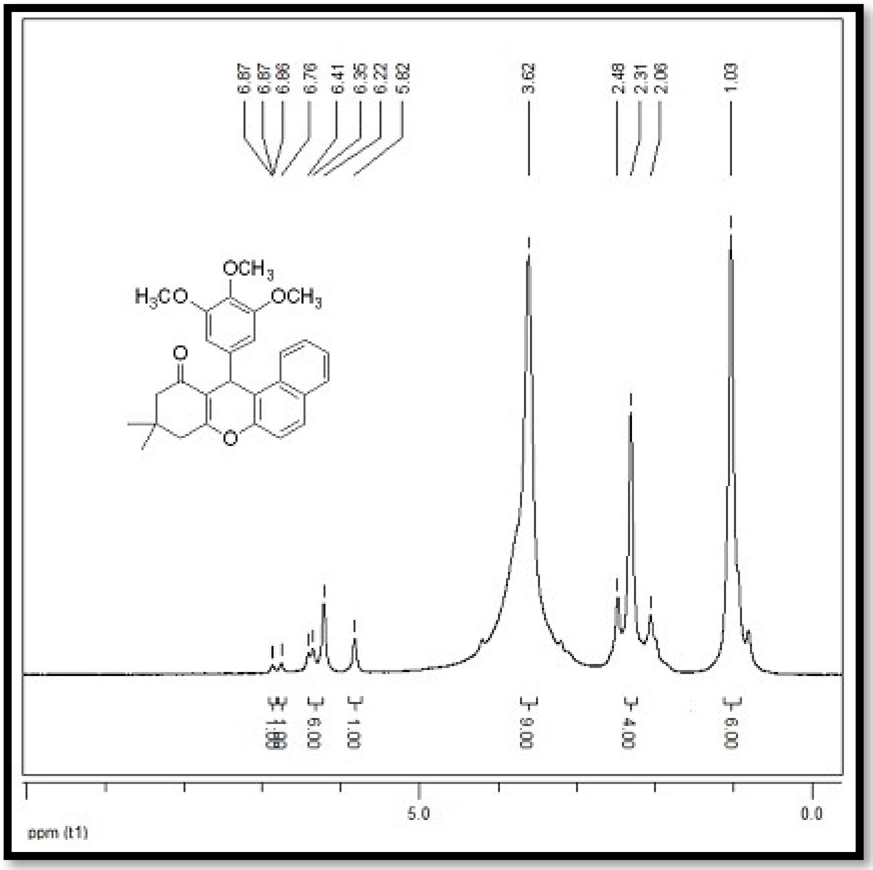

Fig. 15. ^{1}H NMR spectrum of compound 4k.

^{1}H NMR of compound (4l)

This file has been converted from its original format for security purposes. Please use C138DD0810875 as a reference.

***12-(4-hydroxy-3-methoxyphenyl)-9,9-dimethyl-8,9,10,12-tetrahydro-11H-benzo[a]xanthen-11-one (4l)*:** Yield: 98%. M,p. = 194-197 ˚C. ^{1}H NMR (250 MHz, DMSO-d_6): δ = 1.00 (s, 6H, $2CH_3$), 2.28 (brs, 4H, $2CH_2$), 3.84 (brs, 3H, OCH_3), 5.72 (s, 1H, CH), 6.50 (brs, 9H, H-Ar), 8.59 (brs, 1H, H-Ar).

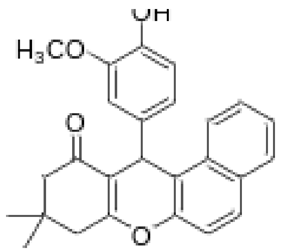

4l

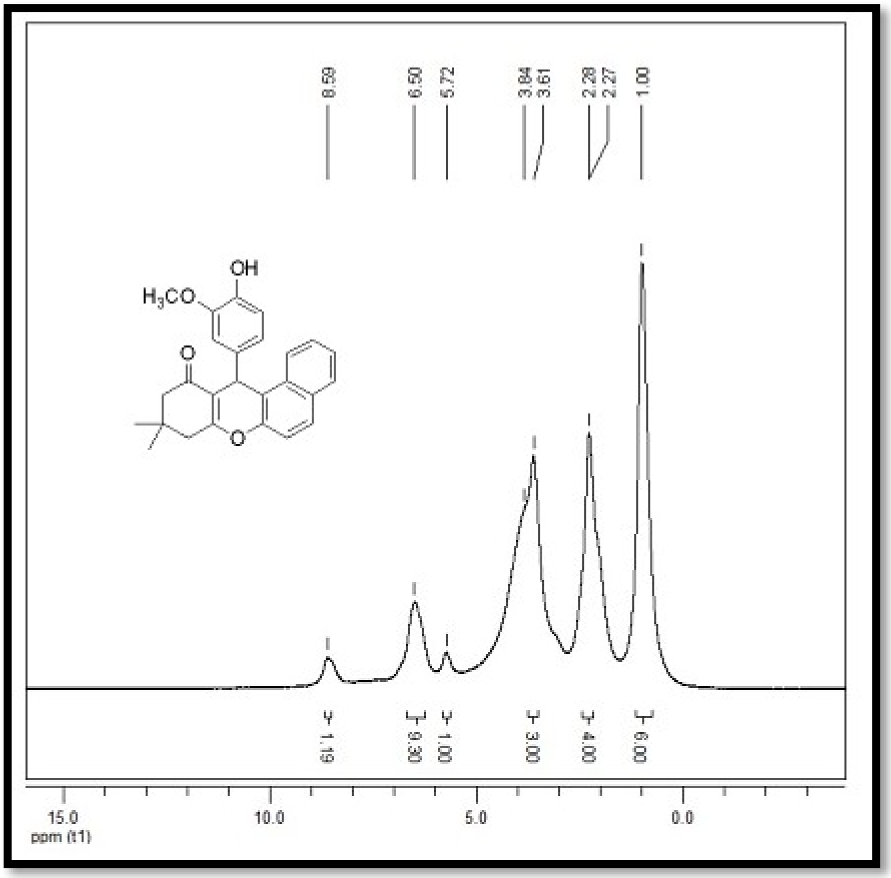

Fig. 16. ^{1}H NMR spectrum of compound 4l.

This file has been converted from its original format for security purposes. Please use C138DD0810875 as a reference.
